# Reporting of Adverse Events in Published and Unpublished Studies of Health Care Interventions: A Systematic Review

**DOI:** 10.1371/journal.pmed.1002127

**Published:** 2016-09-20

**Authors:** Su Golder, Yoon K. Loke, Kath Wright, Gill Norman

**Affiliations:** 1 Department of Health Sciences, University of York, York, United Kingdom; 2 Norwich Medical School, University of East Anglia, Norwich, United Kingdom; 3 Centre for Reviews and Dissemination (CRD), University of York, York, United Kingdom; 4 School of Nursing, Midwifery & Social Work, University of Manchester, Manchester, United Kingdom; Stanford University School of Medicine, UNITED STATES

## Abstract

**Background:**

We performed a systematic review to assess whether we can quantify the underreporting of adverse events (AEs) in the published medical literature documenting the results of clinical trials as compared with other nonpublished sources, and whether we can measure the impact this underreporting has on systematic reviews of adverse events.

**Methods and Findings:**

Studies were identified from 15 databases (including MEDLINE and Embase) and by handsearching, reference checking, internet searches, and contacting experts. The last database searches were conducted in July 2016. There were 28 methodological evaluations that met the inclusion criteria. Of these, 9 studies compared the proportion of trials reporting adverse events by publication status.

The median percentage of published documents with adverse events information was 46% compared to 95% in the corresponding unpublished documents. There was a similar pattern with unmatched studies, for which 43% of published studies contained adverse events information compared to 83% of unpublished studies.

A total of 11 studies compared the numbers of adverse events in matched published and unpublished documents. The percentage of adverse events that would have been missed had each analysis relied only on the published versions varied between 43% and 100%, with a median of 64%. Within these 11 studies, 24 comparisons of named adverse events such as death, suicide, or respiratory adverse events were undertaken. In 18 of the 24 comparisons, the number of named adverse events was higher in unpublished than published documents. Additionally, 2 other studies demonstrated that there are substantially more types of adverse events reported in matched unpublished than published documents. There were 20 meta-analyses that reported the odds ratios (ORs) and/or risk ratios (RRs) for adverse events with and without unpublished data. Inclusion of unpublished data increased the precision of the pooled estimates (narrower 95% confidence intervals) in 15 of the 20 pooled analyses, but did not markedly change the direction or statistical significance of the risk in most cases.

The main limitations of this review are that the included case examples represent only a small number amongst thousands of meta-analyses of harms and that the included studies may suffer from publication bias, whereby substantial differences between published and unpublished data are more likely to be published.

**Conclusions:**

There is strong evidence that much of the information on adverse events remains unpublished and that the number and range of adverse events is higher in unpublished than in published versions of the same study. The inclusion of unpublished data can also reduce the imprecision of pooled effect estimates during meta-analysis of adverse events.

## Abstract 2

In a systematic review, Su Golder and colleagues study the completeness of adverse event reporting, mainly associated with pharmaceutical interventions, in published articles as compared with other information sources.

## Author Summary

**Why Was This Study Done?:**

- Research on medical treatments provides information on the efficacy of such treatments, and on side effects.
- The balance between efficacy and side effects is important in assessing the overall benefit of a new treatment.
- How much information on the side effects of medical treatments that is currently not published in journal articles is not known.

**What Did the Researchers Do and Find?:**

- We searched several databases and other sources, and found 28 studies that provided information on the amount of data on side effects in published journal articles as compared to other sources (such as websites, conferences, and industry-held data).
- The 28 studies found that a lower percentage of published studies than unpublished studies contain information on side effects of treatments.
- A lower number of side effects are generally reported in published than unpublished studies, and a wider range of named side effects are reported in unpublished than published studies.
- Including unpublished data in research leads to more precise conclusions.

**What Do These Findings Mean?:**

- These findings suggest that researchers should search beyond journal publications for information on side effects of treatments.
- These findings also support the need for the drug industry to release full data on side effects so that a complete picture can be given to health professionals, policy makers, and patients.

## Introduction

Adverse events (AEs) are harmful or undesirable outcomes that occur during or after the use of a drug or intervention but are not necessarily caused by it [1]. Information on the adverse events of health care interventions is important for decision-making by regulators, policy makers, health care professionals, and patients. Serious or important adverse events may occur rarely and, consequently, systematic reviews and meta-analyses that synthesize harms data from numerous sources (potentially involving both published and unpublished datasets) can provide greater insights.

The perceived importance of systematic reviews in assessing harms is exemplified by the growing number of such reviews published over the past few years. The Database of Abstracts of Reviews of Effects (DARE) includes 104 reviews of adverse events published in 2010 and 344 in 2014 [2–4]. We have previously noted that systematic reviewers, in line with current guidance [5–7], are increasingly conducting broader searches that include unpublished sources, such as theses and dissertations, conference proceedings, trial registries, and information provided by authors or industry [2–4]. This is despite the difficulties in obtaining and incorporating unpublished data into systematic reviews [8].

Nevertheless, there remains considerable uncertainty about the extent of unpublished or industry data on adverse events beyond that reported in the published literature [9,10]. In 2010, Golder and colleagues found that risk estimates of adverse drug effects derived from meta-analyses of unpublished data and meta-analyses of published data do not differ [9]. However, only 5 studies were included in this previous review [9].

Serious concerns have emerged regarding publication bias or selective omission of outcomes data, whereby negative results are less likely to be published than positive results. This has important implications for evaluations of adverse events because conclusions based on only published studies may not present a true picture of the number and range of the events. The additional issue of poor reporting of harms in journal articles has also been repeatedly highlighted [11–13]. The problem of missing or unavailable data is currently in the spotlight because of campaigns such as AllTrials (www.alltrials.net), the release of results in trial registries (through the 2007 Food and Drug Administration Amendment Act) [14], and increased access to clinical study reports (CSRs) [15]. In addition, reporting discrepancies, including omission of information, have recently been identified between studies published as journal articles and the corresponding unpublished data [16].

These emerging concerns indicate that publication and reporting biases may pose serious threats to the validity of systematic reviews of adverse events. Hence, we aimed to estimate the potential impact of additional data sources and the extent of unpublished information when conducting syntheses of adverse events. This present methodological review updates previous work [9] by focusing on quantifying the amount of unpublished adverse events data as compared to published data, and assessing the potential impact of including unpublished adverse events data on the results of systematic reviews.

## Methods

The review protocol describing the methods to be employed was approved by all the authors before the study was initiated, and only one protocol amendment was made, whereby the risk of bias was changed to take into account the fact that we separately considered matched and unmatched cohorts (see S2 Text).

### Inclusion Criteria

Any type of evaluation was considered eligible for inclusion in this review if it compared information on adverse events of health care interventions according to publication status (i.e., published versus unpublished). “Published” articles were generally considered to be manuscripts that are found in peer-reviewed journals. “Unpublished” data could be located through any other avenue (for example, from regulatory websites, trial registries, industry contact, or personal contact) and included “grey literature,” whereby grey literature is defined as print or electronic information not controlled by commercial or academic publishers. Examples of grey literature include government reports, working papers, press releases, theses, and conference proceedings.

All health care interventions were eligible (such as drug interventions, surgical procedures, medical devices, dentistry, screening, and diagnostic tests). Eligible articles were those that quantified the reporting of adverse events—in particular, the number, frequency, range, or risk of adverse events. This included instances in which the same study was compared in both its published and unpublished format (i.e., “matched comparisons”), such as a journal article and a CSR, as well as evaluations that compared adverse events outcomes from different sets of unrelated published and unpublished sources addressing the same question (i.e., “unmatched comparisons”). We selected these types of evaluations as they would enable us to judge the amount of information that would have been missed if the unpublished data were not available, and to assess the potential impact of unpublished data on pooled effect size in evidence synthesis of harms.

In summary, the PICO was as follows: P (Population), any; I (Intervention), any; C (Comparisons), published and unpublished data and/or studies; O (Outcomes), number of studies, patients, or adverse events, types of adverse events, or adverse event odds ratios and/or risk ratios.

### Exclusion Criteria

We were primarily concerned with the effects of interventions under typical use in a health care setting. We therefore did not consider the broader range of effects, such as intentional and accidental poisoning (i.e., overdose), drug abuse, prescribing and/or administration errors, or noncompliance.

We did not include systematic reviews that had searched for and compared case reports in both the published format and from spontaneous reporting systems (such as the Yellow Card system in the United Kingdom). It is apparent that more case reports can be identified via spontaneous reporting than through the published literature, and a different pattern of reporting has already been established [17]. Moreover, the numbers of people exposed to the intervention in either setting are not known.

We did not restrict our inclusion criteria by year of study or publication status. Although our search was not restricted by language, we were unable to include those papers for which a translation is not readily available or those in a language unknown to the authors.

### Search Methods

A wide range of sources were searched to reflect the diverse nature of publishing in this area. The databases covered areas such as nursing, research methodology, information science, and general health and medicine as well as grey literature sources such as conferences, theses, and the World Wide Web. In addition, handsearching was carried out of key journals, an existing extensive bibliography of unpublished studies, and all years of the Cochrane library. The sources searched are listed in S3 Text. The original searches were conducted in May 2015, with updated searches in July 2016.

Other methods for identifying relevant articles included reference checking of all the included articles and related systematic reviews, citation searching of any key papers on Google Scholar and Web of Science, and contacting authors and experts in the field.

The methodological evaluations from the previous review were included in the analysis, in addition to the papers identified from the new search [9].

The results of the searches were entered into an Endnote library, and the titles and abstracts were sifted independently by two reviewers. Any disagreements were resolved by discussion, and the full articles were retrieved for potentially relevant studies. Full text assessment was then carried out independently by two reviewers and disagreements discussed; when agreement could not be reached, a third independent arbiter was required.

### Search Strategies

The search strategies contained just two facets—“publication status” and “adverse events”—representing the two elements of the review’s selection criteria. Where possible, a database date entry restriction of 2009 onwards was placed on the searches, as this is the date of the last searches carried out [9]. No language restrictions were placed on the searches, although financial and logistical restraints did not allow the translation from all languages. Any papers not included on the basis of language were listed in the table of excluded studies. The MEDLINE search strategy is contained in S4 Text, and this search strategy was translated as appropriate for the search interfaces used for each database.

### Data Extraction

Data were extracted by one reviewer and then all data were checked by another reviewer. Any discrepancies were resolved by discussion or by a third reviewer when necessary. Information was collected on the study design and methodology, interventions and adverse events studied, the sources of published and unpublished data and/or studies, and outcomes reported, such as effect size or number of adverse events.

### Assessment of Methodological Quality

No quality assessment tool exists for these types of methodological evaluations or systematic reviews with an evaluation embedded. The quality assessment criteria were therefore derived in-house by group discussion to reflect a range of possible biases. We assessed validity based on the following criteria:

1 Adequacy of search to identify unpublished and published versions: for instance, were at least two databases searched for the published versions? Incomplete searches would give the impression of either fewer published or unpublished sources in existence.
2 Blinding: was there any attempt to blind the data extractor to which version was published or unpublished?
3 Data extraction: was the data extraction checked or independently conducted when comparing adverse events between the different sources?
4 Definition of publication status: were explicit criteria used to categorise or define unpublished and published studies? For example, unpublished data may consist of information obtained directly from the manufacturers, authors or regulatory agencies. Conversely, a broader definition incorporating “grey literature” may include information from websites, theses or dissertations, trial registries, policy documents, research reports, and conference abstracts, although these are often publically available.
5 External validity and representativeness: did the researchers select a broad-ranging sample of studies (in terms of size, diversity of topics, and spectrum of adverse events) that were reasonably reflective of current literature?

We were aware that published and unpublished data could be compared in different ways. An unconfounded comparison is possible if investigators checked the reported adverse event outcomes in the published article against the corresponding (matching) unpublished version of the same study. In these instances, we assessed validity with the following additional criteria:

6 a. Matching and confirming versions of the same study: for instance, were explicit criteria for intervention, sample size, study duration, dose groups, etc. used? Were study identification numbers used, or were links and/or citations taken from unpublished versions?

Conversely, there are comparisons of adverse events reports between cohorts of unmatched published and unpublished sources, in which investigators synthesise harms data from collections of separate studies addressing the same question. This situation is typically seen within meta-analyses of harms, but we recognize major limitations from a methodological perspective when comparing pooled estimates between published and unpublished studies that are not matched. This stems from the possibility of confounding factors that drive differences in findings between the published and unpublished sources rather than publication status alone. We assessed validity of these comparisons with the following additional criteria:

6 b. Confounding factors in relation to publication status: the results in published sources may differ from those of unpublished sources because of factors other than publication status, such as methodological quality, study design, type of participant, and type of intervention. For instance, did the groups of studies being compared share similar features, other than the difference in publication status? Did the unpublished sources have similar aims, designs, and sample sizes as the published ones? If not, were suitable adjustments made for potentially confounding factors?

We did not exclude any studies based on the quality assessment, as all the identified studies were of sufficient quality to contribute in some form to the analysis.

### Analysis

We considered that there would be different approaches to the data analysis depending on the unit of analysis. We aimed, therefore, to use the following categories of comparison, which are not necessarily mutually exclusive, as some evaluations may provide comparative data in a number of categories:

1. Comparison by number of studies
  Numbers of studies that reported specific types or certain categories of adverse events. This, for instance, may involve identifying the number of published and unpublished studies that report on a particular adverse event of interest (e.g., heart failure) or of a broader category, such as “serious adverse events.”
2. Comparison by adverse events frequencies
  - Number or frequency of adverse events
    How many instances of adverse events were reported (such as the number of deaths reported or number of fracture effects recorded) in the published as compared to unpublished sources?
  - Description of types of adverse events
    How many different types of specific adverse events (such as heart failure, stroke, or cancer) were described in the published as compared to unpublished sources?
3. Comparison by odds ratios or risk ratios
  The impact of adding unpublished sources to the summary estimate—in terms of additional number of studies and participants—as well as the influence on the pooled effect size in a meta-analysis.

In all instances (1 to 3), the published and unpublished sources could be matched (i.e., different versions of the same study) or unmatched (i.e., separate sets of studies on the same topic area).

If pooled risk ratios or odds ratios were presented, we conducted a comparison of the magnitude of treatment effect from published sources versus that of combining all sources (published and unpublished) via a graphical representation.

To avoid double data analysis, where data were presented for all adverse events, serious adverse events, withdrawals because of adverse events, and specific named adverse events, the primary analysis was conducted with the largest group of adverse events (mostly all adverse events or all serious adverse events). In addition, we undertook subgroup analysis with the serious adverse events only.

## Results

**Fig 1:**
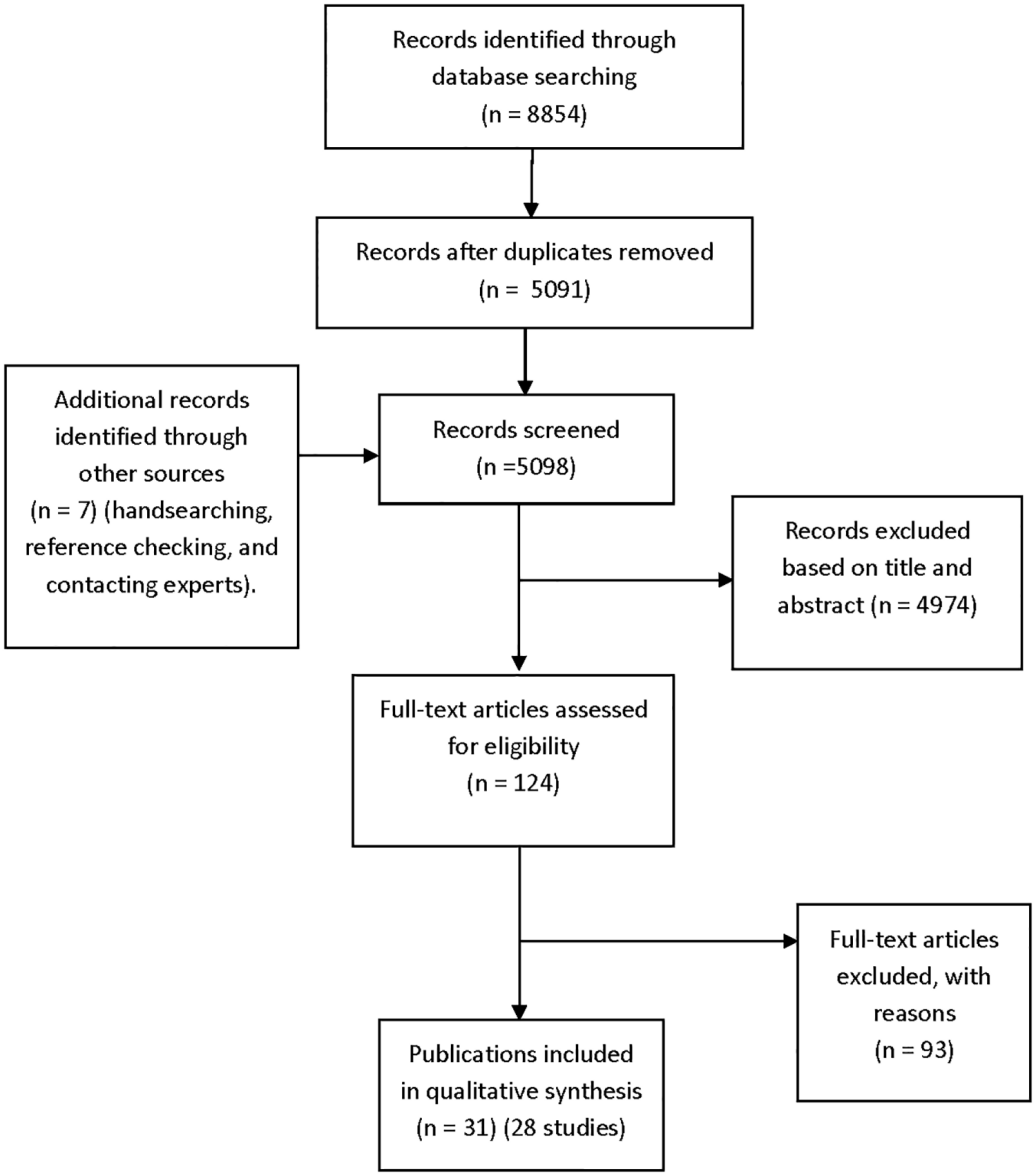
Flow diagram for included studies. Ninety-three articles were excluded based on full-text screening. The excluded studies tended not to include adverse events data or did not present relevant data for both published and unpublished articles (S2 Table).

A total of 4,344 records (6,989 before duplicates were removed) were retrieved from the original database searches in May 2015, and an additional 747 records were identified from the update searches in July 2016. Altogether, 28 studies met the inclusion criteria from 31 publications (Fig 1) (S1 Table) [16,18–47], and 23 studies were not included in our previous review [9].

### Characteristics of Included Studies

The majority of the included studies looked at drug interventions. Only 2 considered a medical device—both of which had a pharmacological component [18,36]—and three looked at all intervention types (although most were still drug interventions) (S3 Table) [22,34,40].

A total of 11 studies considered named adverse events [18,19,24,25,28–31,37,39,42], with 8 looking at both named adverse events and all adverse events [16,18,25,28,31,37,39,42]. All the other studies looked at all adverse events, all serious adverse events, or withdrawals because of adverse events.

The included studies fell into three categories: those which measured the numbers of studies reporting adverse events in published and unpublished sources (9 studies [16,23,25,27,30,34,35,40,43,44]), those which measured the numbers or types of adverse events in published and unpublished sources (13 studies [16,18,22,25–28,32,34,36,38,40,46,47]), and those which measured the difference in effect size of a meta-analysis with and without unpublished sources (11 studies [19–21,24,29,31,33,37,39,41,42,45]). There were 5 studies that contained data for more than one category.

### Summary of Methodological Quality

A total of 26 studies conducted literature searches for the published papers; the other 2 studies either did not report the sources searched [28] or used licensing data (S3 Table) [23]. Although the level of reporting was not always sufficient for a full assessment of the adequacy of the searches, 25 studies used more than one source or approach to searching. Published studies tended to be identified from searches of databases such as PubMed and were defined as studies published in peer-reviewed journals. Unpublished study data were obtained from the manufacturer, investigators, regulatory agencies such as the United States Food and Drug Administration, and trial registries (such as ClinicalTrials.gov or industry registries).

Blinding of researchers to the publication status was not reported in any of the studies. This probably reflects the difficulty of blinding, as it is obvious to data extractors, for example, whether they are looking at an unpublished CSR or a journal paper. In one paper, the authors even refer to the impossibility of blinding. Some form of double data extraction was reported in 22 studies, 3 studies used a single data extractor only [19,24,28] and another 3 studies did not report on this part of the process [23,30,39].

Although many of the studies included any adverse events, the generalisability of the majority of the included studies was limited, as they were all restricted to one drug intervention or one class of drugs (e.g., selective serotonin reuptake inhibitors [SSRIs] or nonsteroidal anti-inflammatory drugs [NSAIDs]).

There were 16 studies that used matched published and unpublished sources. Of these, 8 studies did not describe how they matched unpublished and published articles of the same study [18,25–28,30,36,40]. However, methods such as using the links in ClinicalTrials.gov, clinical trial identifiers, and matching characteristics (including number of participants) were reported in the other studies.

A total of 14 studies compared unmatched published and unpublished sources (2 studies also used matched published and unpublished sources). Although some studies compared the characteristics of published and unpublished sources, only 1 study controlled for confounding factors (S3 Table) [29].

### Comparisons of the Number of Matched Published and Unpublished Studies Reporting Adverse Events

**Fig 2:**
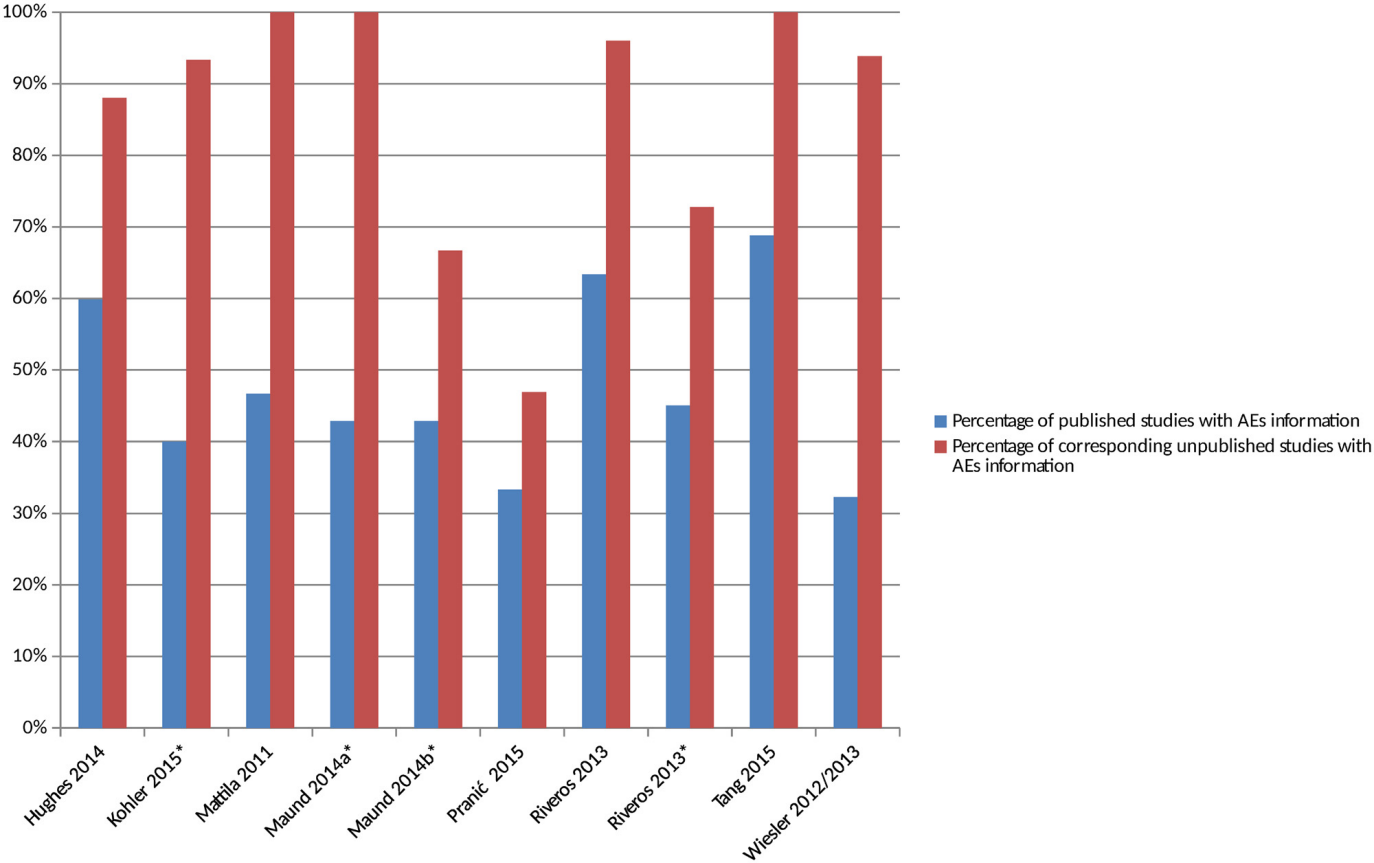
Percentage of matched published and unpublished studies with adverse event information. *Classified adverse events information as either “completely reported” versus “incompletely reported.” Incompletely reported adverse events could lack numerical data or include only selected adverse events, for example. Maund 2014a [16] and Maund 2014b [16] compare published trials to registry reports and clinical study reports (CSRs), respectively. Riveros 2013 [35] compares trials with number of adverse events reported and classifies adverse events information as either “completely reported” or “incompletely reported.”

There were 8 included studies (from 9 publications and representing 10 comparisons) that compared the number of studies reporting adverse events in the published and matched unpublished documents (S4 Table) [16,25,27,30,34,35,40,43,44]. The percentage of studies reporting adverse events was higher in all cases in the unpublished versions than the published versions (Fig 2). The median percentage of published sources with adverse events information was 46% compared to 95% of the corresponding unpublished documents. A similar pattern emerged when the analysis was restricted to serious adverse events (S1 Fig). When types of unpublished studies were compared, a higher number of CSRs tended to contain adverse events data than registry reports [16,43,44].

### Comparisons of the Number of Unmatched Published and Unpublished Studies Reporting AEs

**Fig 3:**
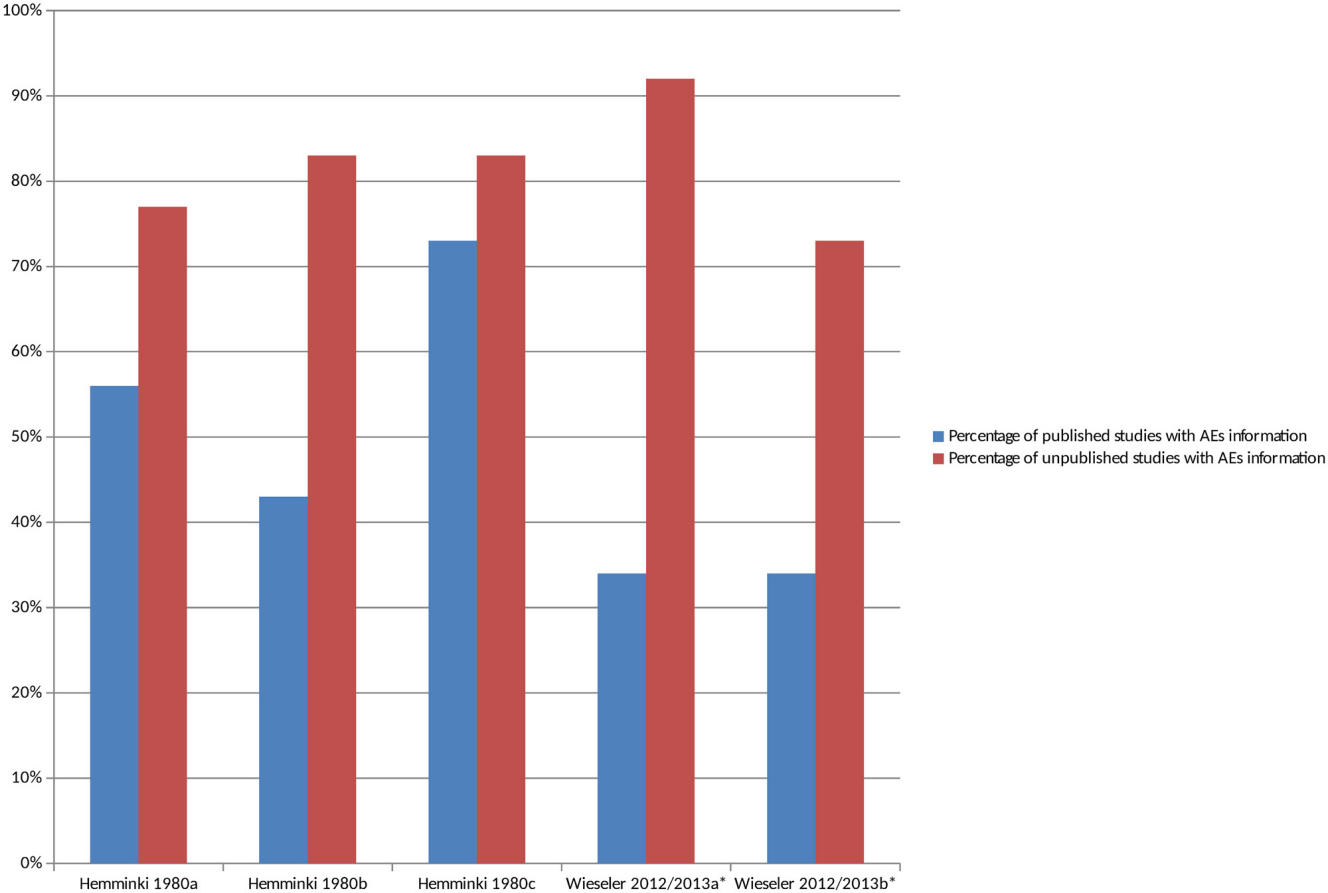
Percentage of unmatched published and unpublished sources with adverse event information. *Classified adverse events information as either “completely reported” versus “incompletely reported.” Incompletely reported adverse events could lack numerical data or include only selected adverse events, for example. Hemminki 1980a [23], 1980b [23], and 1980c [23] compare different drugs in different countries. Wieseler 2012 [44] and 2013a [43] and Wieseler 2012 [44] and 2013b [43] compare published sources with CSRs and registry reports, respectively.

There were 2 studies that compared the number of studies reporting adverse events in unmatched published and unpublished sources (S4 Table) [23,43,44]. The percentage of unpublished studies with information on adverse events was higher overall in all cases than the percentage of published studies with information on adverse events (Fig 3), and this pattern persisted when the analysis was restricted to serious adverse events (S2 Fig). The median percentage of published studies with adverse events information was 43% compared to 83% of unpublished documents. It was interesting to note in the 1 study that compared different types of unpublished sources to published sources that the percentage of CSRs with adverse event information was higher than the registry reports [43,44].

### Comparisons of the Number of AEs in Matched Published and Unpublished Sources

**Fig 4:**
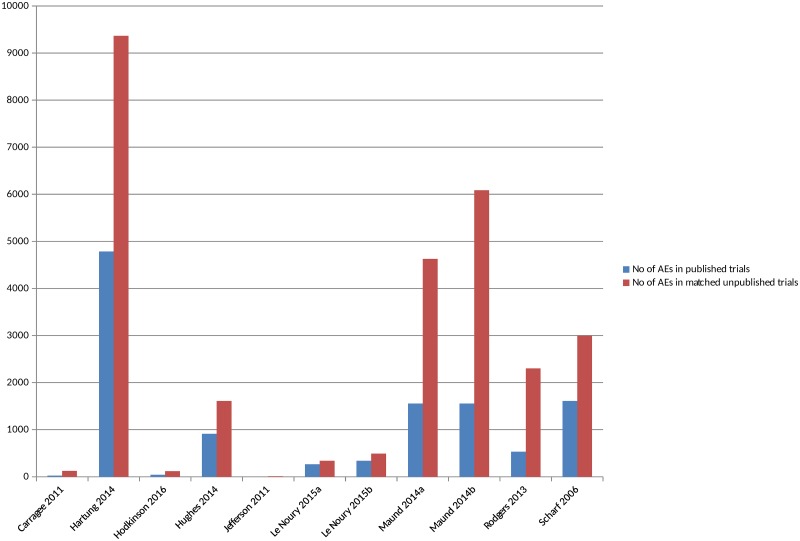
Adverse events in matched published and unpublished sources. In circumstances where multiple AEs were presented, the largest category is included (for example, all AEs or all SAEs). Pranić 2015 [34] and Tang 2015 [40] are excluded from the figure because, although the authors state that they identified a higher number of adverse events in unpublished sources than published, the actual figures were not presented. Le Noury 2015a [28] and 2015b [28] compare AEs for different drugs. Maund 2014a [16] and Maund 2014b [16] compare published trials to registry reports and CSRs, respectively. In Jefferson 2011 [26], the number of adverse events is small (10 events) and therefore does not show up in Fig 4 given the scale of the *y*-axis.

There were 11 included studies that compared the actual numbers of adverse events, such as number of deaths or number of suicides [16,18,22,25,26,28,34,36,38,40,46] (S4 Table) (although in 2 studies the numbers were not explicitly reported). All the studies, without exception, identified a higher number of all or all serious adverse events in the unpublished versions compared to the published versions (Fig 4) (S3 Fig). In some instances, no adverse events, or no adverse events of a particular type, were reported in the published version of a trial but were present in the unpublished version, whilst the discrepancies were noted to be large in other instances. The 1 study that compared different types of unpublished sources to published sources also identified a higher number of adverse events from CSRs than from registry reports [16].

**Fig 5:**
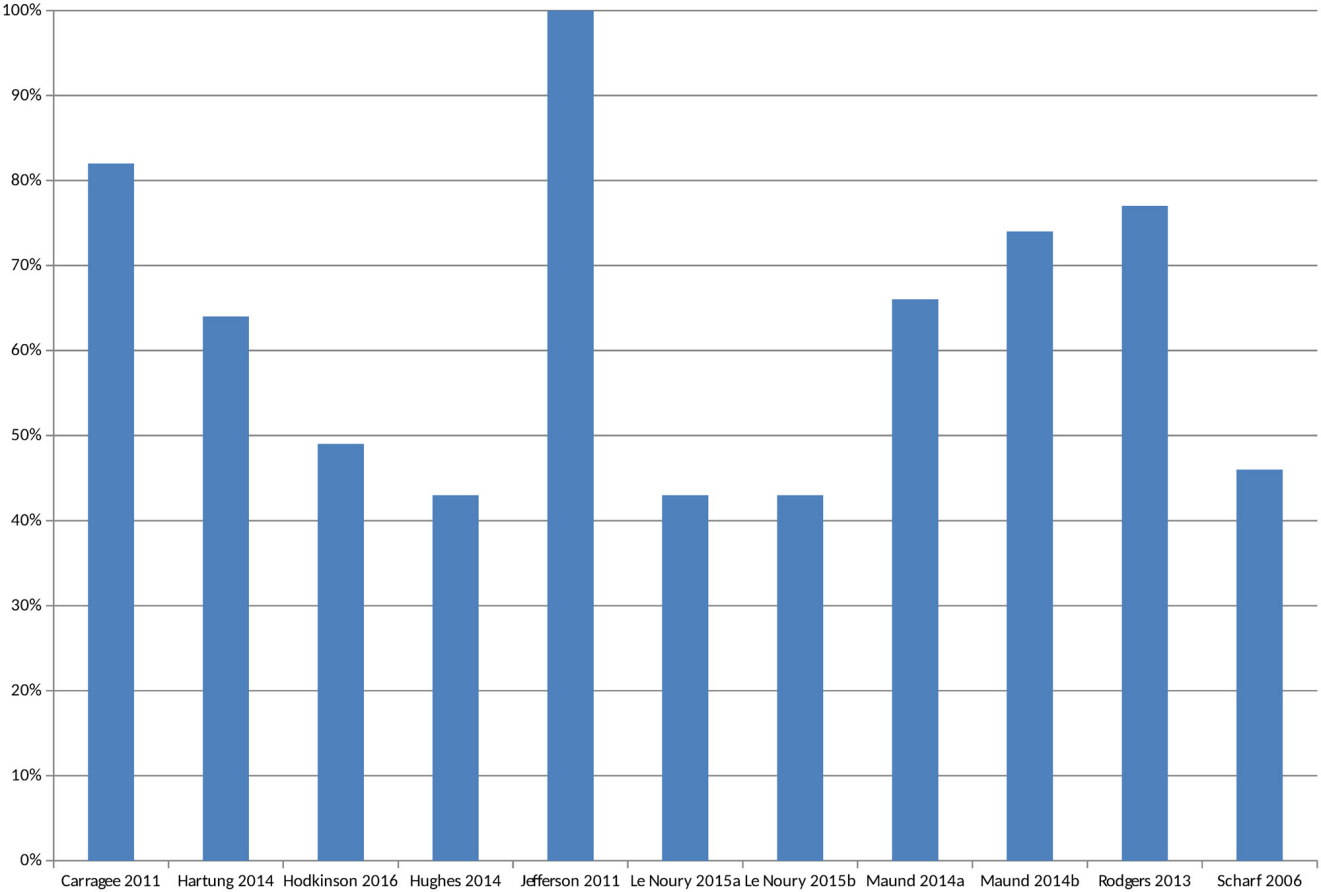
Percentage of adverse events missed without matched unpublished data. In circumstances where multiple AEs were presented, the largest category is included in Fig 5 (for example, all AEs or all SAEs). Pranić 2015 [34] and Tang 2015 [40] are excluded because, although they state that they identified more adverse events in unpublished sources than published, the actual figures were not presented. Le Noury 2015a [28] and 2015b [28] compare AEs for different drugs. Maund 2014a [16] and Maund 2014b [16] compare published trials to registry reports and CSRs, respectively.

The overall percentage of adverse events that would have been missed had an analysis relied only on the published versions of studies varied between 43% and 100%, with a median of 64% (mean 62%) (Fig 5). The percentage of serious adverse events that would have been missed ranged from 2% to 100% (S4 Fig).

Some studies reported the number of adverse events of specific named outcomes, such as death, suicide, or infection, or the numbers of specific categories of adverse events, such as urogenital, cardiovascular, and respiratory adverse events. There were 24 such comparisons, and, in 18 cases, the numbers of adverse events reported in the unpublished documentation were higher than in the trial publications. In three instances, the numbers of adverse events were the same [18,25,28], whilst in another three instances (all from the same study), the numbers of gastrointestinal and/or digestive or neurological and/or nervous system adverse events were higher in the published trial [28].

### Comparison of the Number of Different Types of Specific AEs in Matched Published and Unpublished Sources

There were 2 studies that compared how many different types of specific named adverse events were in matched published and unpublished sources. Pang (2011) found that 67.6% and 93.3% of the serious or fatal adverse events, respectively, in the company trial reports were not fully listed in the published versions [32]. Similarly, Kohler (2015) found that 67% of the different types of adverse events found in unpublished data were not fully detailed in the published versions [27].

### Comparisons of the AEs in Unmatched Published and Unpublished Sources

Only 1 study compared the numbers of adverse events from unmatched published and unpublished articles (in addition to matched articles) (S4 Table) [25]. Although there were fewer unpublished data sources than published data sources, the total number of serious adverse events was still higher in the unpublished sources. Analysis of specific adverse events comprising the total number of suicide ideations, attempts, or injury, homicidal ideations, and psychiatric symptoms were all higher in the unpublished sources. Conversely, the numbers of instances of death and suicide were higher in the published sources.

### Studies that Evaluated Pooled Effect Sizes with Published Studies Alone, as Compared to Published and Unpublished Studies Combined

There were 11 included studies reporting 28 meta-analyses that looked at magnitude of the pooled risk estimate—i.e., odds ratio or risk ratio for adverse events—with unpublished data and published data or a combination of published and unpublished data (S4 Table) [19–21,24,29,31,33,37,39,41,42,45]. Of these 28 meta-analyses, there were 20 instances where risk estimates were pooled for all published and unpublished studies as compared to published studies only. We also found 24 instances where it was possible to evaluate pooled estimates from published alone versus unpublished alone.

Although 2 studies looked at a variety of conditions and treatments, 3 of the 11 studies looked at antidepressants and 2 at cardiovascular treatments, while the other drugs examined were for conditions such as diabetes, eye problems, pain, and menopausal symptoms.

### Amount of Additional Data in the Meta-Analysis from Inclusion of Unpublished Studies

**Fig 6:**
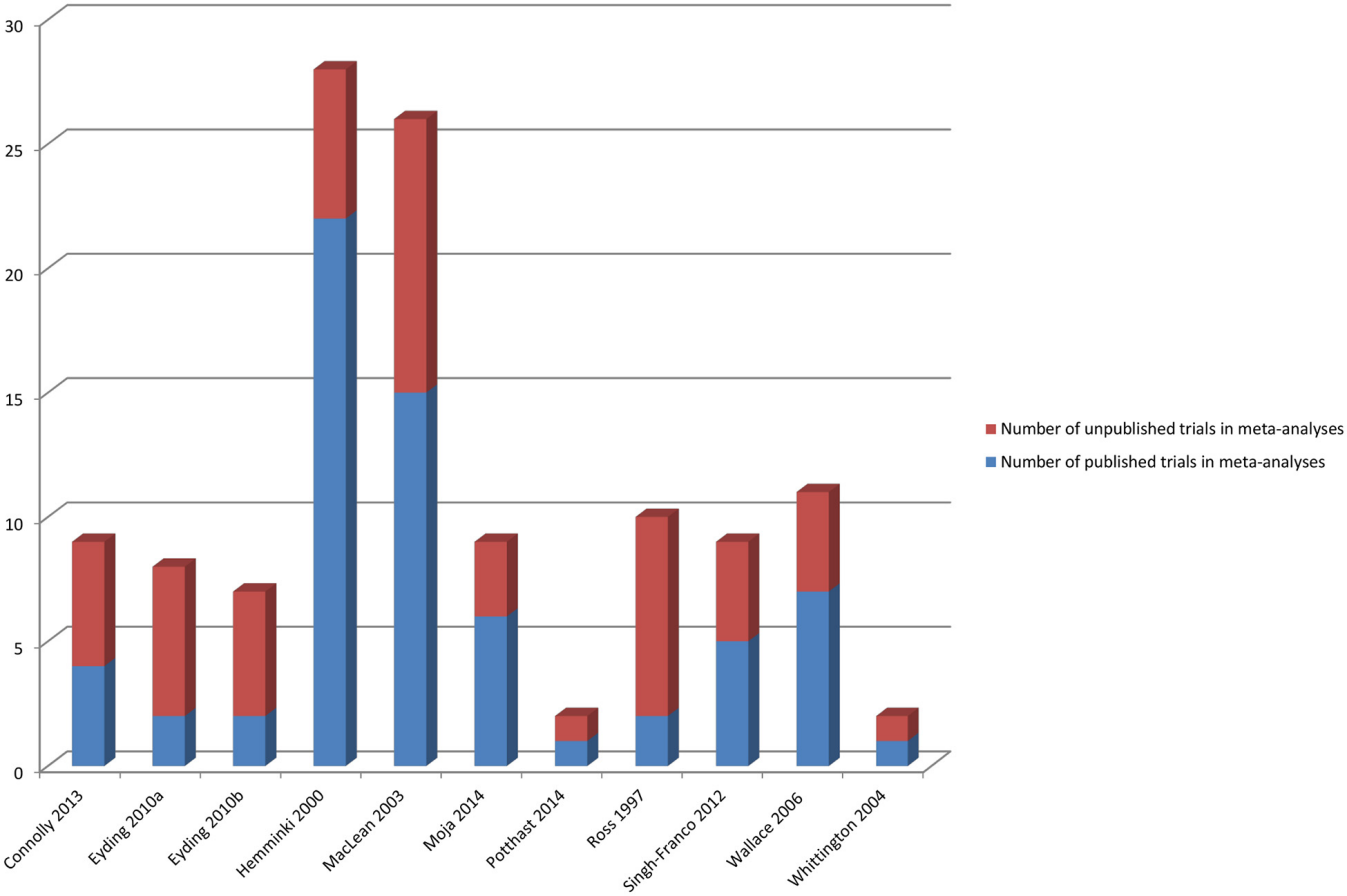
Number of published and unpublished sources in the meta-analyses. Eyding 2010a [20] examines studies of reboxetine versus placebo and Eyding 2010b [20] examines studies of reboxetine versus selective serotonin reuptake inhibitors (SSRIs).

**Fig 7:**
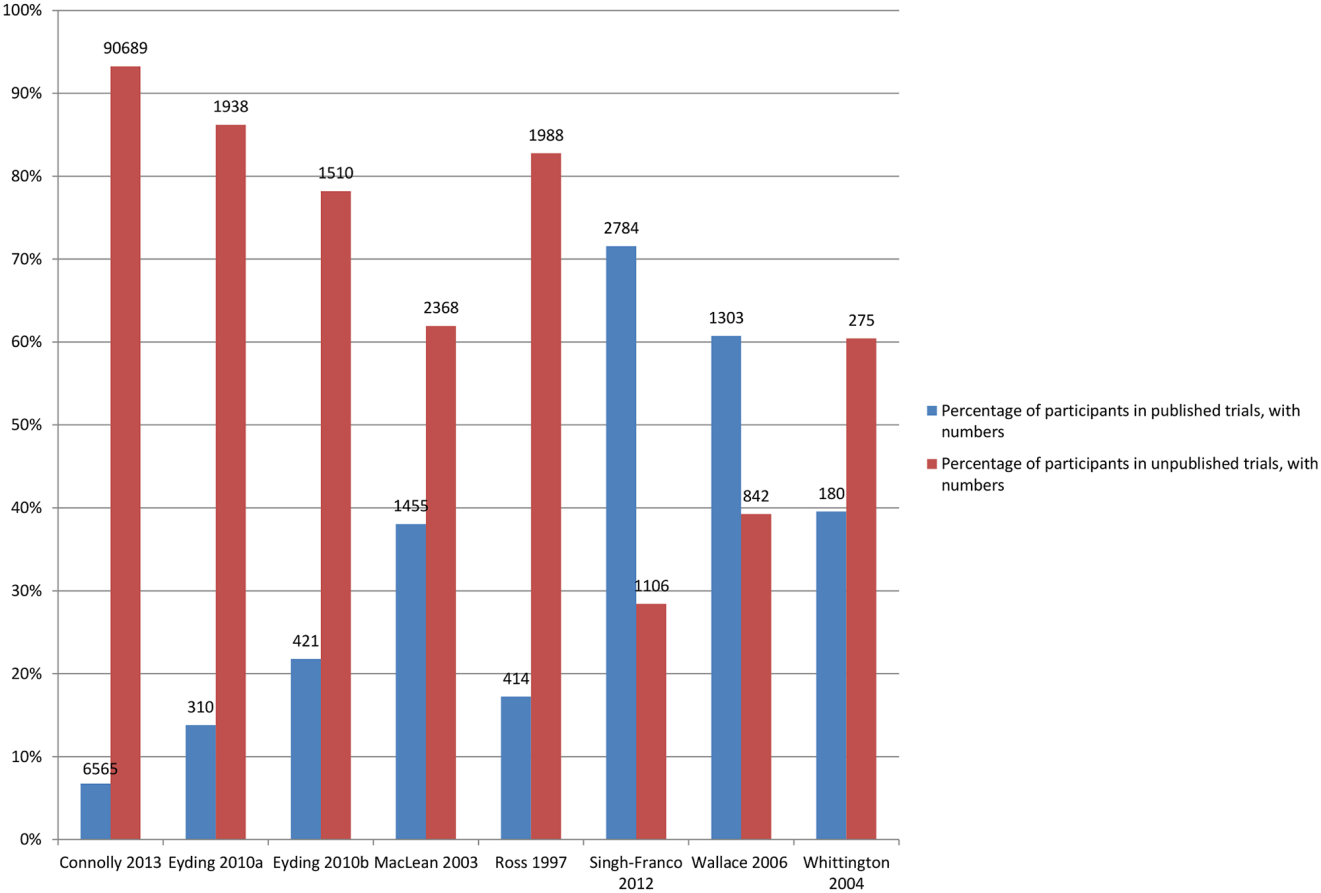
Percentage of participants in published and unpublished studies in the meta-analyses. Eyding 2010a [20] examines studies of reboxetine versus placebo and Eyding 2010b [20] examines studies of reboxetine versus selective serotonin reuptake inhibitors (SSRIs).

Fig 6 shows the number of additional trials available for the meta-analyses, and Fig 7 shows the number of additional participants covered by the meta-analyses when unpublished data were used.

### Results of Comparisons of ORs and/or RRs

**Fig 8:**
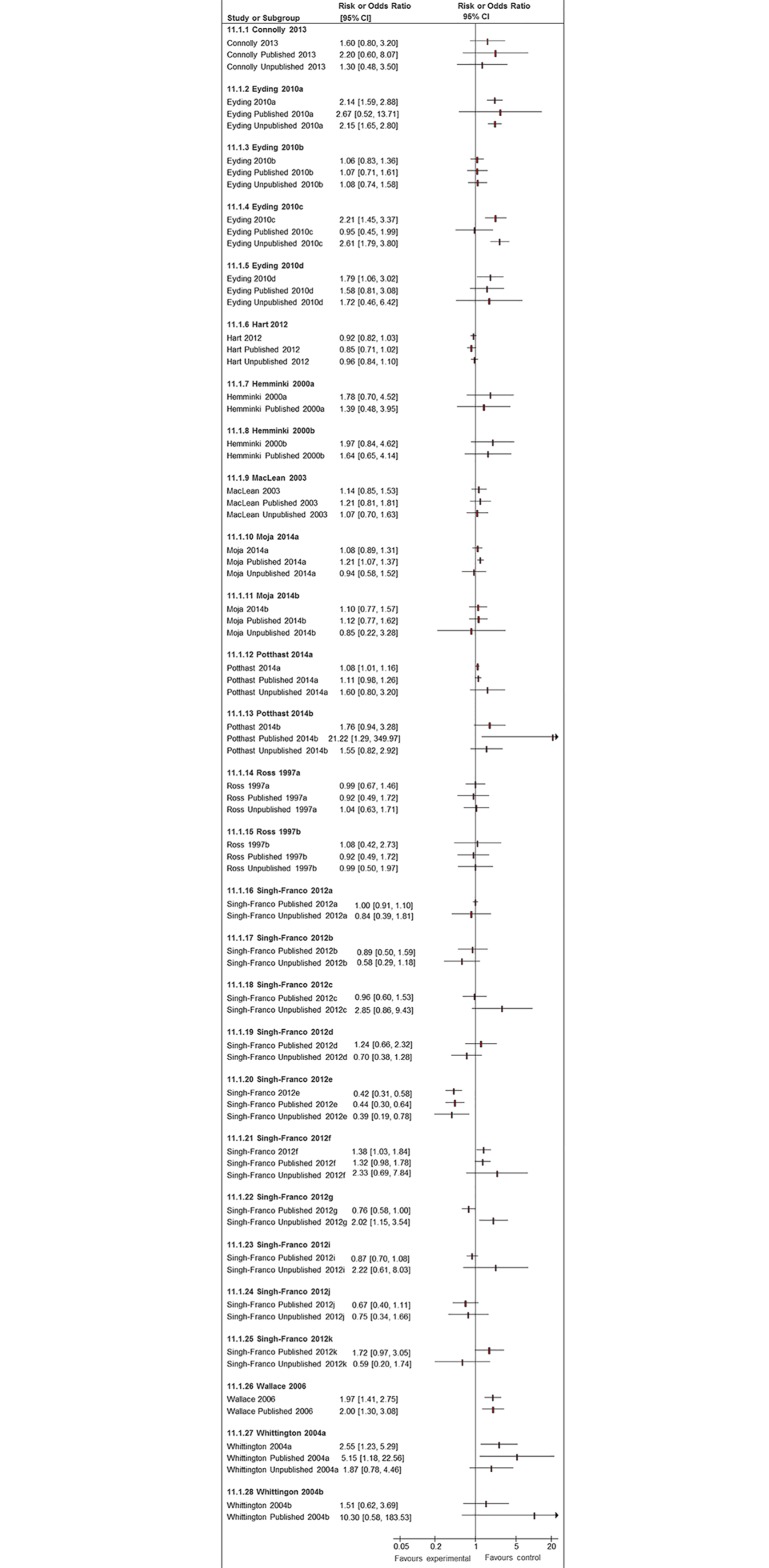
Forest plots comparing pooled estimates for adverse events from all studies combined (published and unpublished) against published and unpublished studies alone. There were also two systematic reviews identified in Potthast 2014 [33] in which unpublished additional trial data led to a new comparison not reported in the systematic reviews, which could not be represented here.

Adverse events are often rare or of low frequency, and we found that meta-analyses of published studies alone tended to yield effect estimates with very broad 95% confidence intervals. In Fig 8, we present the 20 meta-analyses for which we were able to compare pooled estimates with published studies alone against those when unpublished and published data where pooled. The inclusion of unpublished data increased the precision of the pooled estimate, with narrower 95% confidence intervals in 15 of these 20 pooled analyses (Fig 8). Given the breadth of the 95% confidence intervals of the pooled estimates from the published data, we did not identify many instances where addition of unpublished data generated diametrically different effect estimates or 95% confidence intervals that did not overlap with the original estimates from published data only. There were 3 analyses where a nonstatistically significant pooled estimate of increased risk (published studies alone) became statistically significant after unpublished data was added (Eyding 2010a [20], Eyding 2010c [20], and Singh-Franco 2012e [39]). Conversely, the opposite occurred in Moja 2010a [31], in which a significantly increased risk estimate from published studies was rendered nonsignificant after addition of unpublished data.

In Fig 8, we also present 24 meta-analyses in which pooled estimates based on published studies can be compared to pooled estimates using unpublished studies. In eight instances, point estimates from the published and unpublished pooled analysis showed opposing directions of effect; however, the direction and magnitude of the difference varies and is not consistent.

## Discussion

The 28 studies included in our review give an indication as to the amount of data on adverse events that would have been missed if unpublished data were not included in assessments. This is in terms of the number of adverse events, the types of adverse events, and the risk ratios of adverse events. We identified serious concerns about the substantial amount of unpublished adverse events data that may be difficult to access or “hidden” from health care workers and members of the public. Incomplete reporting of adverse events within published studies was a consistent finding across all the methodological evaluations that we reviewed. This was true whether the evaluations were focused on availability of data on a specific named adverse event, or whether the evaluations aimed to assess general categories of adverse events potentially associated with an intervention. Our findings suggest that it will not be possible to develop a complete understanding of the harms of an intervention unless urgent steps are taken to facilitate access to unpublished data.

The extent of “hidden” data has important implications for clinicians and patients who may have to rely on (incomplete) published data when making evidence-based decisions on benefit versus harms. Our findings suggest that poor reporting of harms data, selective outcome reporting, and publication bias are very serious threats to the validity of systematic reviews and meta-analyses of harms. In support of this, there are case examples of systematic reviews that arrived at a very different conclusion once unpublished data were incorporated into the analysis. These examples include the Cochrane review on a neuraminidase inhibitor, oseltamivir (Tamiflu) [48], and the Health Technology Assessment (HTA) report on reboxetine and other antidepressants [15]. Although the process of searching for unpublished data and/or studies can be resource-intensive—requiring, for example, contact with industry, experts, and authors and searches of specialist databases such as OpenGrey, Proquest Dissertations and Theses, conference databases, or websites and trial registries—we strongly recommend that reviews of harms make every effort to obtain unpublished trial data. In addition, review authors should aim to specify the number of studies and participants for which adverse outcome data were unavailable for analysis. The overall evidence should be graded downwards in instances where substantial amounts of unpublished data were not accessible.

Our review demonstrates the urgent need to progress towards full disclosure and unrestricted access to information from clinical trials. This is in line with campaigns such as AllTrials, which are calling for all trials to be registered and the full methods and results to be reported. We are starting to see major improvements, however, in the availability of unpublished data based on initiatives of the European Medicines Agency (EMA) (since 2015, EMA policy on publication of clinical data has meant the agency has been releasing clinical trial reports on request, and proactive publication is expected by September 2016), European law (the Clinical Trial Regulation), the FDA Amendments Act of 2007—which requires submission of trial results to registries—and pressure from Cochrane to fully implement such policies. Although improvements have been made in the accessibility of data, there are still major barriers and issues to contend with. Researchers are still failing to obtain company data, and even when data are released, which can be after a lengthy process, they can be incomplete [49,50], and can have low compliance with results reporting in ClinicalTrials.gov [35,51,52]. The release of CSRs is particularly positive given that this review suggests that CSRs may contain more information than other unpublished sources, such as registry reports.

The discrepant findings in numbers of adverse events reported in the unpublished reports and the corresponding published articles are also of great concern. Systematic reviewers may not know which source contains the most accurate account of results and may be making choices based on inadequate or faulty information. Many studies stated that they were unclear as to why the discrepancies exist, whilst others referred to incomplete reporting or nonreporting, changing the prespecified outcomes analysed (post hoc analysis), or different iterations of the process of aggregating raw data. It is not clear if the differences stemmed from slips in attention and errors, or whether the peer review process led to changes in the data analysis. Journal editors and readers of systematic reviews should be aware that a tendency to overestimate benefit and underestimate harm in published papers can potentially result in misleading conclusions and recommendations [53].

One major limitation that we are aware of is the difficulty of demonstrating (even with meta-analytic techniques) that inclusion of unpublished data leads to changes in direction of effect or statistical significance in pooled estimates of rare adverse events. The meta-analysis case examples here represent only a small number amongst thousands of meta-analyses of harms, and it would be entirely inappropriate to draw conclusions regarding any potential lack of impact on statistical significance of the pooled estimate from inclusion of unpublished data.

Nevertheless, the available examples clearly demonstrate that availability of unpublished data leads to a substantially larger number of trials and participants in the meta-analyses. We also found that the inclusion of unpublished data often leads to more precise risk estimates (with narrower 95% confidence intervals), thus representing higher evidence strength according to the GRADE (Grades of Recommendation, Assessment, Development and Evaluation) classification, in which strength of evidence is downgraded if there is imprecision [54]. Finally, we do not believe that it would ever be possible for systematic reviewers to judge beforehand whether addition of unpublished data may or may not lead to change in pooled effect estimates, and therefore it would be greatly preferable to have access to the full datasets.

There are a number of other limitations to this review. The included studies in our review were difficult to identify from the literature, so relevant studies may have been missed. Searches for methodological studies are notoriously challenging because of a lack of standardized terminology and poor indexing. In addition, systematic reviews with some form of relevant analysis embedded in the full text may not indicate this in the title, abstract, or database indexing of the review. Also, those studies identified may suffer from publication bias, whereby substantial differences between published and unpublished data are more likely to be published.

Few studies compared published and unpublished data for nonpharmacological interventions. Yet the importance of publication bias for adverse events of nondrug interventions may be just as important as for drug interventions. Unpublished adverse events data of nondrug interventions may differ from unpublished adverse events data of drugs because of aspects such as regulatory requirements and industry research. To examine the generalisability of our findings, more studies with a range of intervention types are required.

In conclusion, therefore, there is strong evidence that substantially more information on adverse events is available from unpublished than from published data sources and that higher numbers of adverse events are reported in the unpublished than the published version of the same studies. The extent of “hidden” or “missing” data prevents researchers, clinicians, and patients from gaining a full understanding of harm, and this may lead to incomplete or erroneous judgements on the perceived benefit to harm profile of an intervention.

Authors of systematic reviews of adverse events should attempt to include unpublished data to gain a more complete picture of the adverse events, particularly in the case of rare adverse events. In turn, we call for urgent policy action to make all adverse events data readily accessible to the public in a full, unrestricted manner.

## Supporting Information

S1 Fig Percentage of matched published and unpublished studies with serious adverse event information. *Classified adverse events information as either “completely reported” versus “incompletely reported.” Incompletely reported adverse events could lack numerical data or include only selected adverse events, for example. Maund 2014a [16] and Maund 2014b [16] compare published trials to registry reports and Clinical Study Reports (CSRs) respectively. Riveros 2013 [35] compares trials with number of adverse events reported and classifies adverse events information as either “completely reported” or “incompletely reported.”
(TIFF)

S2 Fig Percentage of unmatched published and unpublished sources with serious adverse effect information. *Classified adverse events information as either “completely reported” versus “incompletely reported.” Incompletely reported adverse events could lack numerical data or include only selected adverse events for example. Hemminki 1980a [23], 1980b [23], and 1980c [23] compare different drugs in different countries. Wieseler 2012 [44] and 2013a [43] and Wieseler 2012 [44] and 2013b [43] compare published sources with clinical study reports (CSRs) and registry reports, respectively.
(TIFF)

S3 Fig Serious adverse events in matched published and unpublished sources. In Jefferson 2011 [26], the number of adverse events is small (10 events) and therefore does not show up in Fig 4 given the scale of the *y*-axis.
(TIFF)

S4 Fig Percentage of serious adverse events missed without matched unpublished data. (TIFF)

S1 Table Search results for each database and/or source. (DOCX)

S2 Table Excluded studies. (DOCX)

S3 Table Design and risk of bias. (DOCX)

S4 Table Main outcome measures and results of included studies. (DOCX)

S1 Text PRISMA checklist. (DOC)

S2 Text Review protocol. (DOCX)

S3 Text Data sources searched. (DOCX)

S4 Text MEDLINE search strategy. (DOCX)

## Abbreviations

AE: adverse event
CSR: clinical study report
DARE: Database of Abstracts of Reviews of Effects
EMA: European Medicines Agency
GRADE: Grades of Recommendation, Assessment, Development and Evaluation
HTA: Health Technology Assessment
NSAID: nonsteroidal anti-inflammatory drug
OR: odds ratio
RR: risk ratio
SSRI: selective serotonin reuptake inhibitor
