# Supplementary figures and images for: Reporting of Adverse Events in Published and Unpublished Studies of Health Care Interventions: A Systematic Review
Source: PLoS Med. 2016 Sep 20;13(9):e1002127. doi: 10.1371/journal.pmed.1002127 (PMC5029817; doi:10.1371/journal.pmed.1002127)

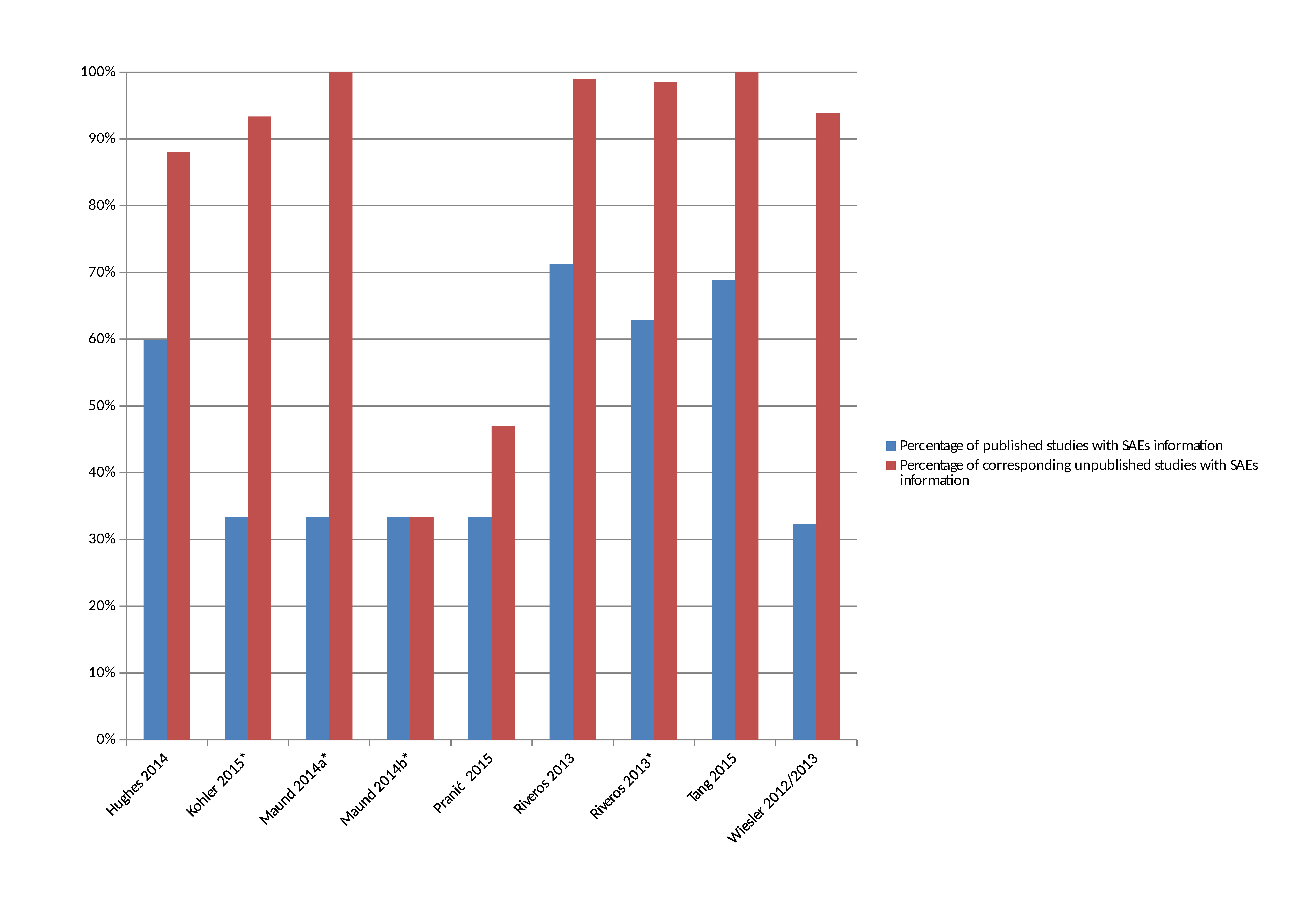

Supplement: S1 Fig — *Classified adverse events information as either “completely reported” versus “incompletely reported.” Incompletely reported adverse events could lack numerical data or include only selected adverse events, for example. Maund 2014a [16] and Maund 2014b [16] compare published trials to registry reports and Clinical Study Reports (CSRs) respectively. Riveros 2013 [35] compares trials with number of adverse events reported and classifies adverse events information as either “completely reported” or “incompletely reported.” (TIFF) [file pmed.1002127.s001.tiff]

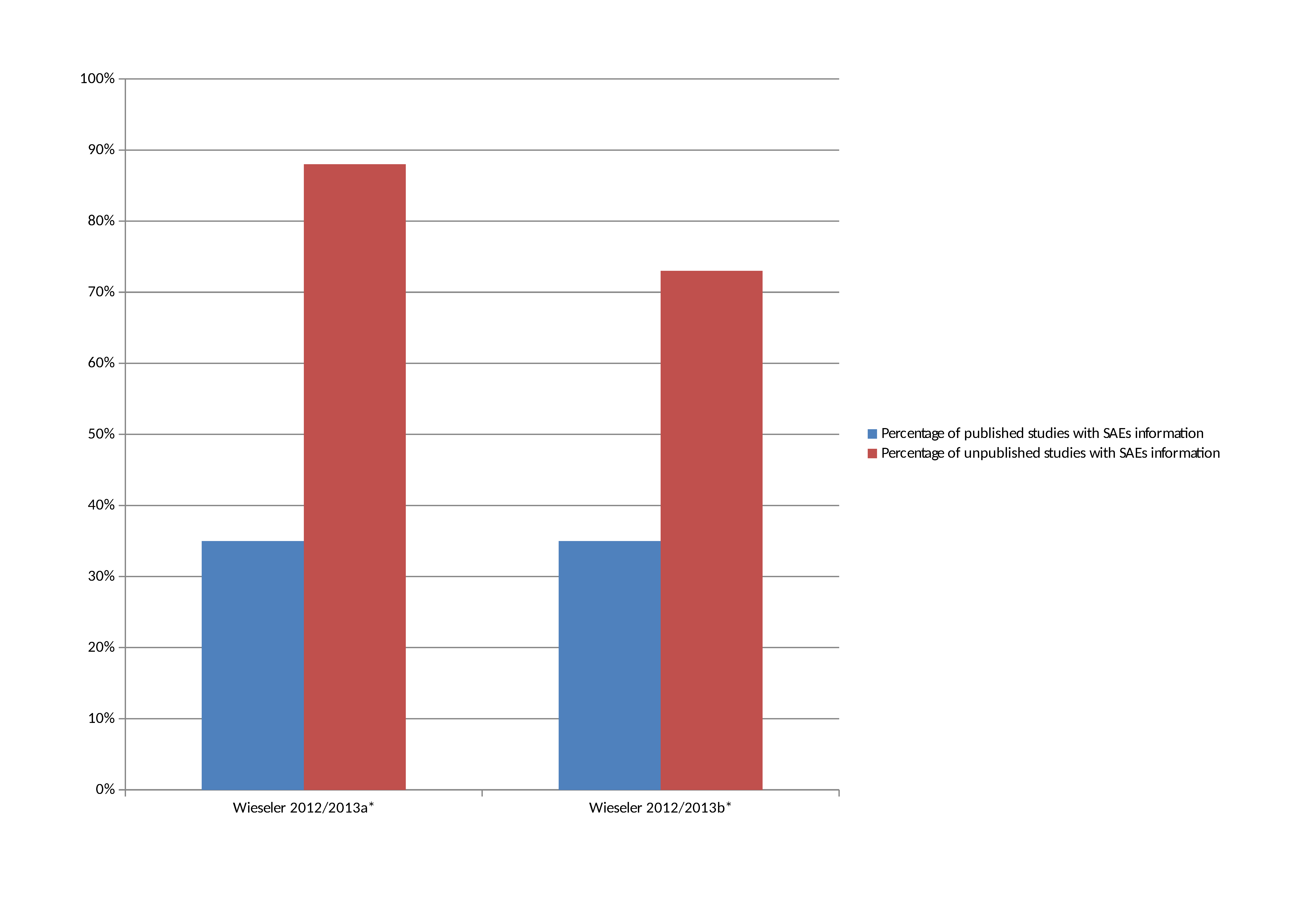

Supplement: S2 Fig — *Classified adverse events information as either “completely reported” versus “incompletely reported.” Incompletely reported adverse events could lack numerical data or include only selected adverse events for example. Hemminki 1980a [23], 1980b [23], and 1980c [23] compare different drugs in different countries. Wieseler 2012 [44] and 2013a [43] and Wieseler 2012 [44] and 2013b [43] compare published sources with clinical study reports (CSRs) and registry reports, respectively. (TIFF) [file pmed.1002127.s002.tiff]

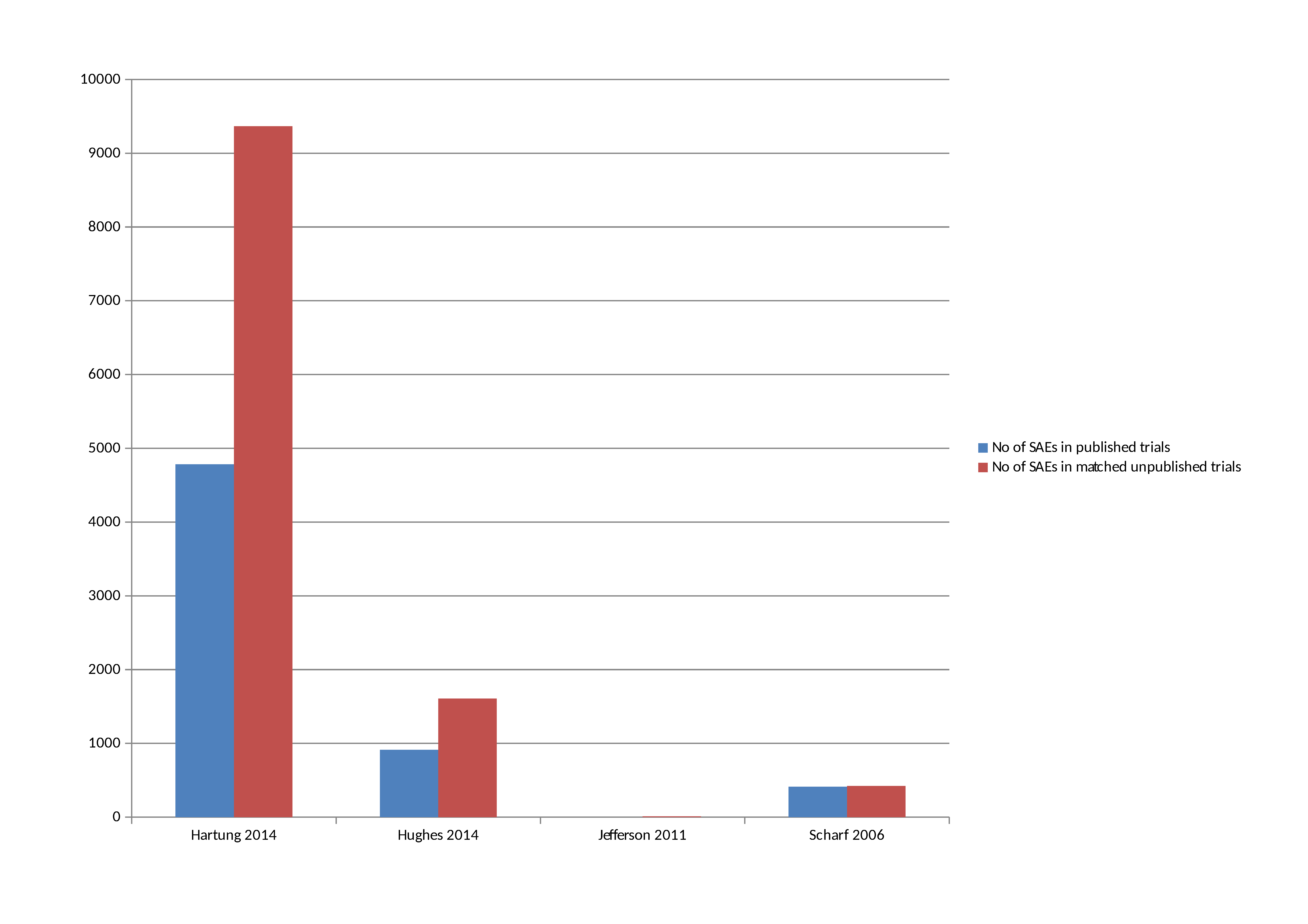

Supplement: S3 Fig — In Jefferson 2011 [26], the number of adverse events is small (10 events) and therefore does not show up in Fig 4 given the scale of the y-axis. (TIFF) [file pmed.1002127.s003.tiff]

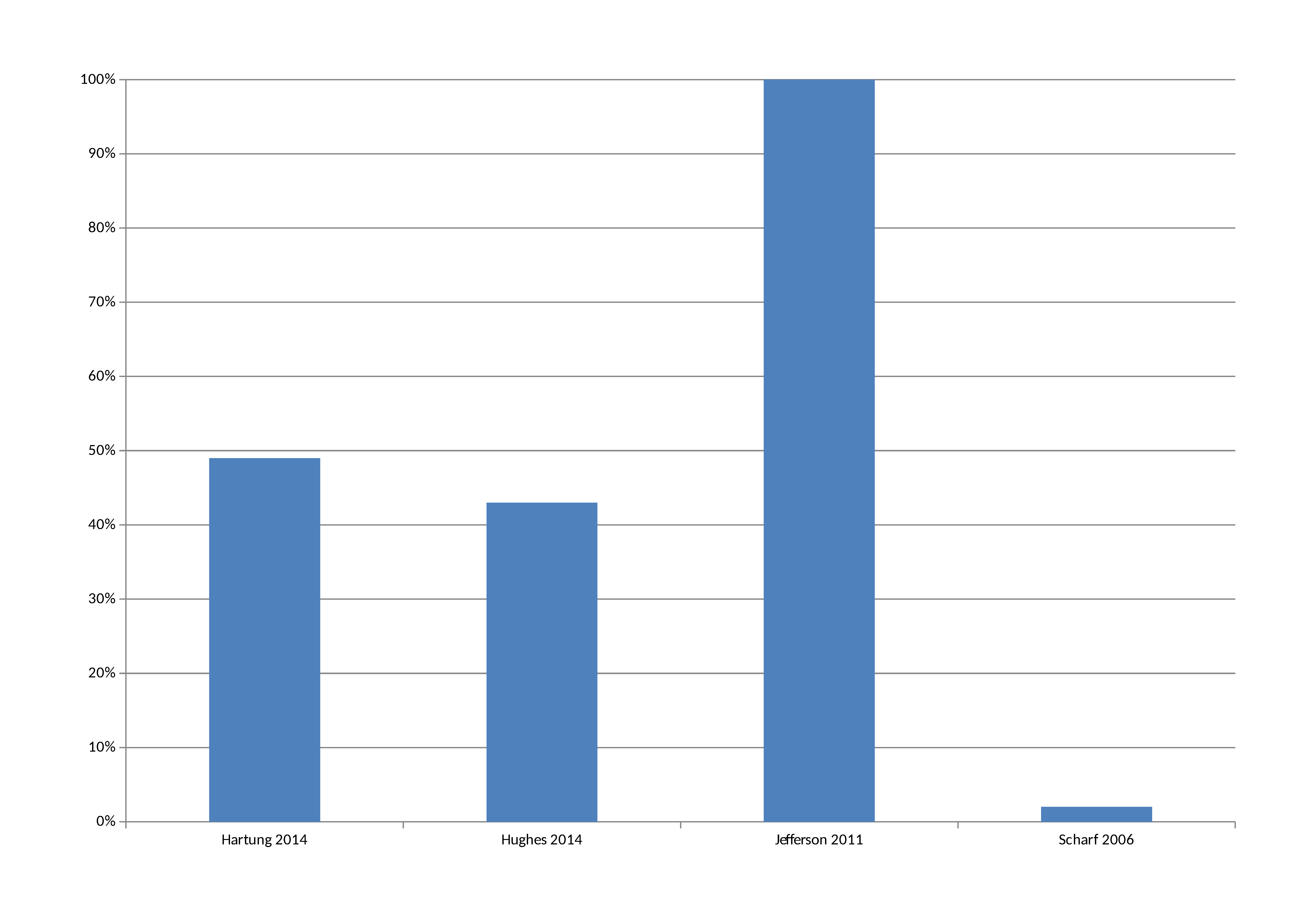

Supplement: S4 Fig — (TIFF) [file pmed.1002127.s004.tiff]
