# Supplementary material for: Reporting of Adverse Events in Published and Unpublished Studies of Health Care Interventions: A Systematic Review
Source: PLoS Med. 2016 Sep 20;13(9):e1002127. doi: 10.1371/journal.pmed.1002127 (PMC5029817; doi:10.1371/journal.pmed.1002127)
Supplement: S1 Table — (DOCX) [file pmed.1002127.s005.docx]

| Database | Interface and version | Original search date | Number of records retrieved | Update search date | Number of records retrieved |
| --- | --- | --- | --- | --- | --- |
| CINAHL Plus | EBSCO (1996 onwards) | 13/05/15 | 492 | 01/07/16 | 60 |
| Cochrane Database of Systematic Reviews (CDSR) – methodology reviews only | The Cochrane Library via Wiley (Issue 5 of 12, May 2015) | 13/05/15 | 0 | 01/07/16 | 0 |
| Cochrane Methodology Register (CMR) | The Cochrane Library via Wiley (Issue 5 of 12, May 2015) | 13/05/15 | 38 | 01/07/16 | 0 |
| Conference Proceedings Citation Index- Science (CPCI-S) | Web of Science (1990-present -LIMITED TO 2007-2015) | 13/05/15 | 87 | 01/07/16 | 17 |
| Embase | OVID (1996 to 2015 Week 19) | 13/05/15 | 1774 | 01/07/16 | 426 |
| Google | Internet | 14/05/15 | 200* | 06/07/16 | 200* |
| Google Scholar | Internet | 14/05/15 | 200* | 06/07/16 | 200* |
| HMIC Health Management Information Consortium | Ovid (1979 to March 2015) | 14/05/15 | 26 | 01/07/16 | 2 |
| MEDLINE and MEDLINE In-process | Ovid (1946 to Present) | 14/05/15 | 1198 | 01/07/16 | 306 |
| OpenGrey | Internet | 14/05/15 | 344 | 01/07/16 | 0 |
| ProQuest Dissertations & Theses: UK & Ireland | Proquest | 14/05/15 | 30 | 01/07/16 | 3 |
| Proquest Library Science and Library and Information Science & Technology Abstracts (LISTA) | Proquest (2007 to current) | 14/05/15 | 6 | 01/07/16 | 9 |
| PsycINFO | Ovid (2002 to May Week 1 2015) | 14/05/15 | 106 | 01/07/16 | 57 |
| Science Citation Index (SCI) | Web of Science (Indexes=SCI-EXPANDED Timespan=1996-2015) | 13/05/15 | 2034 | 01/07/16 | 452 |
| Scopus | Scopus.com | 14/05/15 | 259 | 01/07/16 | 92 |
| Zetoc | Zetoc.jisc.ac.uk | 14/05/15 | 195 | 01/07/16 | 41 |
| TOTAL | | | |  |  |
| Before deduplication | | | 6989 |  | 1865 |
| After deduplication | | | 4344 |  | 1351  (747 after deduplication against original endnote library) |
