# Supplementary material for: Reporting of Adverse Events in Published and Unpublished Studies of Health Care Interventions: A Systematic Review
Source: PLoS Med. 2016 Sep 20;13(9):e1002127. doi: 10.1371/journal.pmed.1002127 (PMC5029817; doi:10.1371/journal.pmed.1002127)
Supplement: S2 Table — (DOCX) [file pmed.1002127.s006.docx]

| **Reference** | **Reason for exclusion** |
| --- | --- |
| Alberti S, Chiesa A, Andrisano C, Serretti A. Insomnia and Somnolence Associated With Second-Generation Antidepressants During the Treatment of Major Depression A Meta-Analysis. J Clin Psychopharmacol. 2015;35(3):296-303. | Review of the literature including unpublished data. No usable data. |
| Amarilyo G, Furst DE, Woo JMP, Li W, Bliddal H, Christensen R, et al. Agreements and Discrepancies between FDA Reports and Journal Papers on Biologic Agents Approved for Rheumatoid Arthritis: A Meta-Research Project. PLoS One. 2016;11(1):13. | Matched study comparison that reports on differences in the way patients were included, events that were counted, and method of analysis. Did not report on missing AE data or differences in pooled estimates, although they did report risk estimates from individual trials. |
| Anderson PA, Hart RA. Adverse events recording and reporting in clinical trials of cervical total disk replacement. Instr Course Lect. 2014;63:287-96. | Compares raw totals without the control arm. |
| Andrade C, Bhakta. S., Singh NM. "Controversy revisited: Selective serotonin reuptake inhibitors in paediatric depression." World J Biol Psychiatry. 2006;7(4):251-60. | Review of the literature including studies which have compared published and unpublished data. No usable data. |
| Benjamin DK, Jr., Smith PB, Sun MJ, Murphy MD, Avant D, Mathis L, [Rodriguez W](http://www.ncbi.nlm.nih.gov/pubmed/?term=Rodriguez%20W%5BAuthor%5D&cauthor=true&cauthor_uid=19996043), [Califf RM](http://www.ncbi.nlm.nih.gov/pubmed/?term=Califf%20RM%5BAuthor%5D&cauthor=true&cauthor_uid=19996043), [Li JS](http://www.ncbi.nlm.nih.gov/pubmed/?term=Li%20JS%5BAuthor%5D&cauthor=true&cauthor_uid=19996043).. Safety and transparency of pediatric drug trials. Arch Pediatr Adolesc Med. 2009;163(12):1080-6. | Not enough quantifiable data. 9/16 of published trials substantially differed in their presentation and interpretation of the data submitted to FDA. Direct quotes presented |
| Bennett DA. FDA: untapped source of unpublished trials. Lancet. 2003;361:1402-3. | Commentary only. |
| [Bielefeldt K](http://www.ncbi.nlm.nih.gov/pubmed/?term=Bielefeldt%20K%5BAuthor%5D&cauthor=true&cauthor_uid=27158546). Adverse events of sacral neuromodulation for fecal incontinence reported to the federal drug administration. [World J Gastrointest Pharmacol Ther.](http://www.ncbi.nlm.nih.gov/pubmed/27158546) 2016 May 6;7(2):294-305. | Published adverse effects and case reports from MAUDE database. |
| Bohlius J, Weingart O, Trelle S, Engert A. Disentangling the data: variation in data submissions from different players and their potential impact on a systematic review. Cochrane Colloquium, Oct 22-26, Melbourne, Australia. 2005. | Conference abstract. No comparison data for unpublished vs published data. States discrepancies in 10/12 comparisons FDA reports/journal publications vs data submitted by pharmaceutical industry for same study. This was due to more AEs in control group in data from pharmaceutical industry but no further details presented. |
| Bolland MJ, Grey A. A comparison of adverse event and fracture efficacy data for strontium ranelate in regulatory documents and the publication record. BMJ Open. 2014;4(10). | Pooled data from regulatory documents for Venous thromboembolism 1.5 (1.1 to 2.1), Pulmonary embolism 1.7 (1.0 to 3.1), Myocardial Infarction 1.6 (1.1 to 2.4) but data on these events were not available for pooled analysis from the primary trial publications |
| Cahill KS, McCormick PC, Levi AD. A comprehensive assessment of the risk of bone morphogenetic protein use in spinal fusion surgery and postoperative cancer diagnosis. J Neurosurg Spine 2015;23(1):86-93. | Review of the literature including unpublished data. No usable data. |
| Carragee E, Weiner B, Hurwitz E. Comparison of adverse events and disclosures in the original rhBMP-2 trials with fda data and subsequent publications. Spine Journal. 2011;1:21S-2S. | Conference abstract. Related to included paper Carragee et al 2011. No information comparing published and unpublished data. |
| [Chahal J](http://www.ncbi.nlm.nih.gov/pubmed/?term=Chahal%20J%5BAuthor%5D&cauthor=true&cauthor_uid=22679295), [Tomescu SS](http://www.ncbi.nlm.nih.gov/pubmed/?term=Tomescu%20SS%5BAuthor%5D&cauthor=true&cauthor_uid=22679295), [Ravi B](http://www.ncbi.nlm.nih.gov/pubmed/?term=Ravi%20B%5BAuthor%5D&cauthor=true&cauthor_uid=22679295), [Bach BR Jr](http://www.ncbi.nlm.nih.gov/pubmed/?term=Bach%20BR%20Jr%5BAuthor%5D&cauthor=true&cauthor_uid=22679295), [Ogilvie-Harris D](http://www.ncbi.nlm.nih.gov/pubmed/?term=Ogilvie-Harris%20D%5BAuthor%5D&cauthor=true&cauthor_uid=22679295), [Mohamed NN](http://www.ncbi.nlm.nih.gov/pubmed/?term=Mohamed%20NN%5BAuthor%5D&cauthor=true&cauthor_uid=22679295), [Gandhi R](http://www.ncbi.nlm.nih.gov/pubmed/?term=Gandhi%20R%5BAuthor%5D&cauthor=true&cauthor_uid=22679295). Publication of sports medicine-related.randomized controlled trials registered in ClinicalTrials.gov. [Am J Sports Med.](http://www.ncbi.nlm.nih.gov/pubmed/22679295) 2012 Sep;40(9):1970-7. | Measures discrepancies in registry primary outcomes and publication. No separate harms data. |
| Chan AW, Krleza-Jeric K, Schmid I, Altman DG Outcome reporting bias in randomized trials funded by the Canadian Institutes of Health research. CMAJ 2004:171 (7): 735–40. | Measures outcome reporting bias by comparing publication to protocol |
| Chan AW, Hrobjartsson A, Haahr MT, Gotzsche PC, Altman DG. Empirical Evidence for selective reporting of outcomes in randomized trials: comparison of protocols to published articles. JAMA 2004:291 (20): 2457–65. | Measures outcome reporting bias by comparing publication to protocol |
| Chang LDS, Chu J, Bero LA, Redberg RF. Selective reporting in trials of high risk cardiovascular devices: cross sectional comparison between premarket approval summaries and published reports. BMJ. 2015;350:h2613. | No usable data. |
| Chatterjee S, Herrmann HC, Wilensky RL, Hirshfeld, J, McCormick D, Frankel DS, Yeh RW, Armstrong EJ, Kumbhani DJ, Giri J. Safety and Procedural Success of Left Atrial Appendage Exclusion With the Lariat Device: A Systematic Review of Published Reports and Analytic Review of the FDA MAUDE Database. JAMA internal medicine 2015;175(7):1104-9. | Published adverse effects and case reports from MAUDE database. |
| Curtis J, Perez-Gutthann S, Suissa S, Napalkov P, Singh N, Thompson L, Porter-Brown B.. Tocilizumab in rheumatoid arthritis: A case study of safety evaluations of a large postmarketing data set from multiple data sources. Semin Arthritis Rheum. 2015;44(4):381-8. | Spontaneous database included published literature, unclear publication status of all sources. |
| De Pablo Lopez de Abechuco I, Aguilar-Jimenez M, Galvez-Mugica MA, Serrano Olmeda M, Revilla-Monaj A, Sanchez-Rodriguez M. Early interruption in clinical trials: Are these results published? Basic and Clinical Pharmacology and Toxicology, 2011;109:34. | Conference abstract. Not enough data. |
| [Derry S](http://www.ncbi.nlm.nih.gov/pubmed/?term=Derry%20S%5BAuthor%5D&cauthor=true&cauthor_uid=22972108), [Moore RA](http://www.ncbi.nlm.nih.gov/pubmed/?term=Moore%20RA%5BAuthor%5D&cauthor=true&cauthor_uid=22972108), [Rabbie R](http://www.ncbi.nlm.nih.gov/pubmed/?term=Rabbie%20R%5BAuthor%5D&cauthor=true&cauthor_uid=22972108). Topical NSAIDs for chronic musculoskeletal pain in adults. [Cochrane Database Syst Rev.](http://www.ncbi.nlm.nih.gov/pubmed/22972108) 2012 Sep 12;(9):CD007400 | Review of the literature including unpublished data. No usable data. |
| Dobson, J.; Whitley, R. J.; Pocock, S.; Monto, A. S.  Oseltamivir treatment for influenza in adults: A meta-analysis of randomised controlled trials. The Lancet 2015;385(9979):1729-37. | Review of the literature including unpublished data. No usable data. |
| Dundar Y, Dodd S, Dickson R, Walley T, Haycox A, Williamson PR. Comparison of conference abstracts and presentations with full-text articles in the health technology assessments of rapidly evolving technologies. Health Technology Assessment. 2006; 10(5) | Compares quality and reporting. No relevant data on AEs |
| Dwan K, Altman DG, Arnaiz JA, Bloom J, Chan AW, Cronin E, et al. Systematic review of the empirical evidence of study publication bias and outcome reporting bias. PLoS ONE, 2008;3(8):e3081. | Systematic review with no relevant data. Included studies checked for references. |
| Dwan K, Altman DG, Cresswell L, Blundell M, Gamble CL, & Williamson PR. (2011). Comparison of protocols and registry entries to published reports for randomised controlled trials. Cochrane Database of Systematic Reviews, (1), MR000031. | Systematic review with no relevant data. Included studies checked for references. |
| Earley A, Lau J, Uhlig K. Haphazard reporting of deaths in clinical trials: a review of cases of ClinicalTrials.gov records and matched publications-a cross-sectional study. BMJ Open. 2013;3(1):9. | Death was either measure of effectiveness or adverse effect. Not presented as adverse effects only. |
| [Ewart R](http://www.ncbi.nlm.nih.gov/pubmed/?term=Ewart%20R%5BAuthor%5D&cauthor=true&cauthor_uid=19901314), [Lausen H](http://www.ncbi.nlm.nih.gov/pubmed/?term=Lausen%20H%5BAuthor%5D&cauthor=true&cauthor_uid=19901314), [Millian N](http://www.ncbi.nlm.nih.gov/pubmed/?term=Millian%20N%5BAuthor%5D&cauthor=true&cauthor_uid=19901314). Undisclosed changes in outcomes in randomized controlled trials: an observational study. [Ann Fam Med.](http://www.ncbi.nlm.nih.gov/pubmed/19901314)2009 Nov-Dec;7(6):542-6. | Investigated the frequency of undisclosed changes between trial registry record and publication. |
| Faundez A; Tournier C; Garcia M; Aunoble S.; Le Huec JC. Bone morphogenetic protein use in spine surgery-complications and outcomes: a systematic review. Int Orthop 2016;40(6):1309-19. | Review of the literature including unpublished data. No usable data. |
| Freedman SB, Uleryk E, Rumantir M, Finkelstein Y. Ondansetron and the Risk of Cardiac Arrhythmias: A Systematic Review and Postmarketing Analysis. Ann Emerg Med. 2014;64(1):19-25. | Case reports from literature ADR databases and grey literature. No denominators. |
| [Gandhi R](http://www.ncbi.nlm.nih.gov/pubmed/?term=Gandhi%20R%5BAuthor%5D&cauthor=true&cauthor_uid=22151841), [Jan M](http://www.ncbi.nlm.nih.gov/pubmed/?term=Jan%20M%5BAuthor%5D&cauthor=true&cauthor_uid=22151841), [Smith HN](http://www.ncbi.nlm.nih.gov/pubmed/?term=Smith%20HN%5BAuthor%5D&cauthor=true&cauthor_uid=22151841), [Mahomed NN](http://www.ncbi.nlm.nih.gov/pubmed/?term=Mahomed%20NN%5BAuthor%5D&cauthor=true&cauthor_uid=22151841), [Bhandari M](http://www.ncbi.nlm.nih.gov/pubmed/?term=Bhandari%20M%5BAuthor%5D&cauthor=true&cauthor_uid=22151841). Comparison of published orthopaedic trauma trials following registration in Clinicaltrials.gov. [BMC Musculoskelet Disord.](http://www.ncbi.nlm.nih.gov/pubmed/?term=comparison+of+published+orthopaedic+trauma+gandhi) 2011 Dec 7;12:278. | Measured consistency between registry record and publication. No separate harms data. |
| Hampton J. Therapeutic fashion and publication bias: The case of anti-arrhythmic drugs in heart attack J R Soc Med. 2015;108(10):418-420. | Discussion paper. |
| [Hannink G](http://www.ncbi.nlm.nih.gov/pubmed/?term=Hannink%20G%5BAuthor%5D&cauthor=true&cauthor_uid=23407296), [Gooszen HG](http://www.ncbi.nlm.nih.gov/pubmed/?term=Gooszen%20HG%5BAuthor%5D&cauthor=true&cauthor_uid=23407296), [Rovers MM](http://www.ncbi.nlm.nih.gov/pubmed/?term=Rovers%20MM%5BAuthor%5D&cauthor=true&cauthor_uid=23407296). Comparison of registered and published primary outcomes in randomized clinical trials of surgical interventions. [Ann Surg.](http://www.ncbi.nlm.nih.gov/pubmed/?term=comparison+of+registered+and+published+primary+hannink) 2013 May;257(5):818-23 | Compared registered and published primary outcomes. No separate harms data. |
| Hart BL, Lundh A, Bero LA. Adding unpublished Food and Drug Administration (FDA) data changes the results of meta-analyses. Poster presentation at the 19th Cochrane Colloquium; 2011 Oct 19-22; Madrid, Spain [abstract]. Cochrane Database of Systematic Reviews, Supplement. 2011 :128-9 | Conference abstract. Related to included paper Hart et al 2012. |
| Hartl R, Joeris A, McGuire R. A. Comparison of the safety outcomes between two surgical approaches for anterior lumbar fusion surgery: anterior lumbar interbody fusion (ALIF) and extreme lateral interbody fusion (ELIF). European Spine Journal 2016;25(5):1484-1521. | Complication rates with and without FDA reports. Looked at inconsistencies between the FDA trial registries, clinical trials.gov, and published reports of trials but no usable data. |
| Heneghan CJ; Onakpoya I; Jones, MA; Doshi P; Del Mar CB; Hama R.; Thompson MJ; Spencer EA.; Mahtani KR; Nunan D; Howick J; Jefferson T. Neuraminidase inhibitors for influenza: A systematic review and meta-analysis of regulatory and mortality data. Health Technology Assessment 2016;20(42):1-242. | Review of the literature including unpublished data. No usable data. |
| Hernandez MV, Sanmarti R, Canete JD. The safety of tumor necrosis factor-alpha inhibitors in the treatment of rheumatoid arthritis. Expert Opin. Drug Saf. 2016;15(5):613-24. | Review of the literature including unpublished data. No usable data. |
| Hill KD; Henderson HT; Hornik CP; Li JS. Paediatric cardiovascular clinical trials: An analysis of ClinicalTrials.gov and the Food and Drug Administration Pediatric Drug Labeling Database. Cardiology in the Young 2015;25(S2):172-80. | Reviews situation and provides summary and analysis of CT.gov and FDA. |
| Hopewell S. Assessing the impact of abstracts from the Thoracic Society of Australia and New Zealand in Cochrane reviews. Respirology, 2003;8:509-512. | No relevant data on AEs. |
| Howland RH. What you see depends on where you're looking and how you look at it: publication bias and outcome reporting bias. Journal of Psychosocial Nursing & Mental Health Services 2011:49(8): 13-5. | Discussion paper. |
| Howland RH. Publication bias and outcome reporting bias: Agomelatine as a case example. Journal of Psychosocial Nursing and Mental Health Services 2011:49(9):11-4. | Discussion paper. |
| Huić M, Marušić M, Marušić A. Completeness and Changes in Registered Data and Reporting Bias of Randomized Controlled Trials in ICMJE Journals after Trial Registration Policy. [PLoS One.](http://www.ncbi.nlm.nih.gov/pubmed/21957485) 2011;6(9):e25258. | Compared published and registry data. No separate harms data. |
| Huynh L, Scherer R, Ervin A, Dickersin K. Primary outcomes reported in abstracts and ClinicalTrials.gov - do they agree? Oral presentation at the 19th Cochrane Colloquium; 2011 Oct 19-22; Madrid, Spain | Conference abstract. Not enough data. |
| Jefferson T, Jones M, Doshi P, Del Mar C. Neuraminidase inhibitors for preventing and treating influenza in healthy adults: systematic review and meta-analysis. BMJ, 2009;339:b5106. | Carries out sensitivity analysis by excluding unpublished trials. |
| Jefferson T; Jones MA; Doshi P; Del Mar CB; Hama R; Thompson MJ; Spencer EA; Onakpoya I; Mahtani K. R; Nunan D; Howick J; Heneghan CJ. Neuraminidase inhibitors for preventing and treating influenza in healthy adults and children. Cochrane Database Syst Rev 2014;4:CD008965 | Review of the literature including unpublished data. No usable data. |
| [Jones CW](http://www.ncbi.nlm.nih.gov/pubmed/?term=Jones%20CW%5BAuthor%5D&cauthor=true&cauthor_uid=22503374), [Platts-Mills TF](http://www.ncbi.nlm.nih.gov/pubmed/?term=Platts-Mills%20TF%5BAuthor%5D&cauthor=true&cauthor_uid=22503374). Quality of registration for clinical trials published in emergency medicine journals. [Ann Emerg Med.](http://www.ncbi.nlm.nih.gov/pubmed/22503374)2012;60(4):458-64.e1. | Measured consistency between registered and published outcomes. |
| Jorgensen AW, Tendal B, Gotzsche PC. The robustness of results on weight loss in trials on sibutramine - a comparison of results from unpublished study reports with the corresponding published reports. Oral presentation at the Joint Cochrane and Campbell Colloquium; 2010 Oct 18-22; Keystone, Colorado. | Conference abstract. No relevant adverse effects data |
| Jureidini JN, McHenry LB, Mansfield PR. Clinical trials and drug promotion: selective reporting of study 329. International Journal of Risk and Safety in Medicine, 2008;20:73-81. | Single case report of study 329. |
| Kaiser T, Wieseler B. Impact of unpublished data from industry sponsored trials on drug assessments [abstract]. XIV Cochrane Colloquium; 2006 October 23-26; Dublin, Ireland. | Conference abstract. No relevant adverse effects data |
| Kaiser T, Kerekes MF, Wieseler B. Industry clinical trials registries as a data source for systematic reviews. Oral presentation at the 17th Cochrane Colloquium; 2009 Oct 11-14, Singapore | Conference abstract. No relevant adverse effects data |
| Kaczmarek MC, Duong UT, Ware RS, Lambert SB, Kelly HA. The risk of fever following one dose of trivalent inactivated influenza vaccine in children aged >6 months to <36 months: a comparison of published and unpublished studies. Vaccine. 2013;31(46):5359-65. | Compares raw totals without the control arm |
| [Killeen S](http://www.ncbi.nlm.nih.gov/pubmed/?term=Killeen%20S%5BAuthor%5D&cauthor=true&cauthor_uid=23732270), [Sourallous P](http://www.ncbi.nlm.nih.gov/pubmed/?term=Sourallous%20P%5BAuthor%5D&cauthor=true&cauthor_uid=23732270), [Hunter IA](http://www.ncbi.nlm.nih.gov/pubmed/?term=Hunter%20IA%5BAuthor%5D&cauthor=true&cauthor_uid=23732270), [Hartley JE](http://www.ncbi.nlm.nih.gov/pubmed/?term=Hartley%20JE%5BAuthor%5D&cauthor=true&cauthor_uid=23732270), [Grady HL](http://www.ncbi.nlm.nih.gov/pubmed/?term=Grady%20HL%5BAuthor%5D&cauthor=true&cauthor_uid=23732270). Registration rates, adequacy of registration, and a comparison of registered and published primary outcomes in randomized controlled trials published in surgery journals. [Ann Surg.](http://www.ncbi.nlm.nih.gov/pubmed/23732270) 2014;259(1):193-6. | Measured discrepancies between registry records and publications for primary outcome. No separate harms data. |
| Kmietowicz Z. Unpublished studies hold twice as much data on drugs as those in the public domain. BMJ. 2013;347(7929):5-5. | Editorial on included paper by Wieseler et al 2013. |
| Korevaar DA; Hooft L. The danger of unpublished trial results. Ned. Tijdschr. Geneeskd. 2014;158(19) | Non-english. Discussion paper. |
| Kraus CN, Burnstead BW. The Safety Record of Fusidic Acid in Non-US Markets: A Focus on Skin Infections. Clin Infect Dis. 2011;52:S527-37. | Case reports from vigibase and the literature. No denominators. |
| LaValley M, Lo G, Hunter D, Felson D. Use of the USA Food and Drug Administration web site for clinical trial information from approved therapies. 12th Cochrane Colloquium: Bridging the Gaps; 2004 Oct 2-6; Ottawa, Ontario, Canada | Conference abstract. No relevant adverse effects data |
| Law MR, Kawasumi Y, Morgan SG. Despite law, fewer than one in eight completed studies of drugs and biologics are reported on time on ClinicalTrials.gov. Health Aff (Millwood). 2011;30(12):2338-45. | Study on the timing of reporting |
| Liebeskind DS, Kidwell CS, Sayre JW, Saver JL. Evidence of publication bias in reporting acute stroke clinical trials. Neurology. 2006;67(6):973-9 | Uses efficacy and harm interchangeably so failure of benefit is considered as harm. Unclear what the authors mean by ‘proportion of harmful outcomes’. |
| Liebeskind DS, Lee M. Evidence of bias and the bias of evidence in neurothrombectomy for stroke. Stroke. 2011;42 (3):e317. | Study on level of evidence |
| [Li XQ](http://www.ncbi.nlm.nih.gov/pubmed/?term=Li%20XQ%5BAuthor%5D&cauthor=true&cauthor_uid=24131272), [Yang GL](http://www.ncbi.nlm.nih.gov/pubmed/?term=Yang%20GL%5BAuthor%5D&cauthor=true&cauthor_uid=24131272), [Tao KM](http://www.ncbi.nlm.nih.gov/pubmed/?term=Tao%20KM%5BAuthor%5D&cauthor=true&cauthor_uid=24131272), [Zhang HQ](http://www.ncbi.nlm.nih.gov/pubmed/?term=Zhang%20HQ%5BAuthor%5D&cauthor=true&cauthor_uid=24131272), [Zhou QH](http://www.ncbi.nlm.nih.gov/pubmed/?term=Zhou%20QH%5BAuthor%5D&cauthor=true&cauthor_uid=24131272), [Ling CQ](http://www.ncbi.nlm.nih.gov/pubmed/?term=Ling%20CQ%5BAuthor%5D&cauthor=true&cauthor_uid=24131272). Comparison of registered and published primary outcomes in randomized controlled trials of gastroenterology and hepatology. [Scand J Gastroenterol.](http://www.ncbi.nlm.nih.gov/pubmed/24131272) 2013;48(12):1474-83. | Presents data on primary outcome consistency. No separate results for harms. |
| Li-Kim-Moy J; Yin JK; Rashid H; Khandaker G; King C; Wood N; Macartney KK; Jones C; Booy R. Systematic review of fever, febrile convulsions and serious adverse events following administration of inactivated trivalent influenza vaccines in children. Eurosurveillance 2015;20(24):1-20. | Review of the literature including unpublished data. No usable data. |
| [Liu JP](http://www.ncbi.nlm.nih.gov/pubmed/?term=Liu%20JP%5BAuthor%5D&cauthor=true&cauthor_uid=23864210), [Han M](http://www.ncbi.nlm.nih.gov/pubmed/?term=Han%20M%5BAuthor%5D&cauthor=true&cauthor_uid=23864210), [Li XX](http://www.ncbi.nlm.nih.gov/pubmed/?term=Li%20XX%5BAuthor%5D&cauthor=true&cauthor_uid=23864210), [Mu YJ](http://www.ncbi.nlm.nih.gov/pubmed/?term=Mu%20YJ%5BAuthor%5D&cauthor=true&cauthor_uid=23864210), [Lewith G](http://www.ncbi.nlm.nih.gov/pubmed/?term=Lewith%20G%5BAuthor%5D&cauthor=true&cauthor_uid=23864210), [Wang YY](http://www.ncbi.nlm.nih.gov/pubmed/?term=Wang%20YY%5BAuthor%5D&cauthor=true&cauthor_uid=23864210), [Witt CM](http://www.ncbi.nlm.nih.gov/pubmed/?term=Witt%20CM%5BAuthor%5D&cauthor=true&cauthor_uid=23864210), [Yang GY](http://www.ncbi.nlm.nih.gov/pubmed/?term=Yang%20GY%5BAuthor%5D&cauthor=true&cauthor_uid=23864210), [Manheimer E](http://www.ncbi.nlm.nih.gov/pubmed/?term=Manheimer%20E%5BAuthor%5D&cauthor=true&cauthor_uid=23864210), [Snellingen T](http://www.ncbi.nlm.nih.gov/pubmed/?term=Snellingen%20T%5BAuthor%5D&cauthor=true&cauthor_uid=23864210), [Berman B](http://www.ncbi.nlm.nih.gov/pubmed/?term=Berman%20B%5BAuthor%5D&cauthor=true&cauthor_uid=23864210), [Gluud C](http://www.ncbi.nlm.nih.gov/pubmed/?term=Gluud%20C%5BAuthor%5D&cauthor=true&cauthor_uid=23864210). Prospective registration, bias risk and outcome-reporting bias in randomised clinical trials of traditional Chinese medicine: an empirical methodological study. [BMJ Open.](http://www.ncbi.nlm.nih.gov/pubmed/23864210) 2013;3(7). | Reports inconsistency rates in safety as a methodological component in registered records and publication. No further details. |
| Ma SJ, Xiong YQ, Jiang LN, Chen Q. Risk of febrile seizure after measles-mumps-rubella-varicella vaccine: A systematic review and meta-analysis. Vaccine 2015;33(31):3636-49. | Review of the literature including unpublished data. No usable data. |
| [Mathieu S](http://www.ncbi.nlm.nih.gov/pubmed/?term=Mathieu%20S%5BAuthor%5D&cauthor=true&cauthor_uid=19724045), [Boutron I](http://www.ncbi.nlm.nih.gov/pubmed/?term=Boutron%20I%5BAuthor%5D&cauthor=true&cauthor_uid=19724045), [Moher D](http://www.ncbi.nlm.nih.gov/pubmed/?term=Moher%20D%5BAuthor%5D&cauthor=true&cauthor_uid=19724045), [Altman DG](http://www.ncbi.nlm.nih.gov/pubmed/?term=Altman%20DG%5BAuthor%5D&cauthor=true&cauthor_uid=19724045), [Ravaud P](http://www.ncbi.nlm.nih.gov/pubmed/?term=Ravaud%20P%5BAuthor%5D&cauthor=true&cauthor_uid=19724045). Comparison of registered and published primary outcomes in randomized controlled trials. [JAMA.](http://www.ncbi.nlm.nih.gov/pubmed/19724045) 2009;302(9):977-84. | Compared primary outcomes in trial registries and publication. No separate results for harms. |
| McDonagh MS, [Peterson K](http://www.ncbi.nlm.nih.gov/pubmed/?term=Peterson%20K%5BAuthor%5D&cauthor=true&cauthor_uid=23856190), [Balshem H](http://www.ncbi.nlm.nih.gov/pubmed/?term=Balshem%20H%5BAuthor%5D&cauthor=true&cauthor_uid=23856190), [Helfand M](http://www.ncbi.nlm.nih.gov/pubmed/?term=Helfand%20M%5BAuthor%5D&cauthor=true&cauthor_uid=23856190). US Food and Drug Administration documents can provide unpublished evidence relevant to systematic reviews. Journal of Clinical Epidemiology 2013;66(10): 1071-81. | Not enough data presented separately on harms. Checked references. |
| Meeker AS, Herink MC, Haxby DG, Hartung DM. The safety and efficacy of vortioxetine for acute treatment of major depressive disorder: A systematic review and meta-analysis. Systematic Reviews 2015;4 (1)(21). | Review of the literature including unpublished data. No usable data. |
| Man-Son-Hing M, Wells GAL. Quinine for nocturnal leg cramps: a meta-analysis including unpublished data. J Gen Intern Med. 1998;13(9):600-9. | Did not present meta-analysis of the OR/RR, but just reports raw totals giving the risk estimate for quinine vs control |
| Moncrieff J. Misrepresenting harms in antidepressant trials. BMJ 2016;352:i217 | Editorial. Checked references. |
| Moro PL, Cragan J, Tepper N, Zheteyeva Y, Museru O, Lewis P, Broder K. Enhanced surveillance of tetanus toxoid, reduced diphtheria toxoid, and acellular pertussis (Tdap) vaccines in pregnancy in the Vaccine Adverse Event Reporting System (VAERS), 2011-2015. Vaccine 2016;34(20):2349-53. | Compares published data and reports to the Vaccine Adverse Event Reporting System (VAERS). |
| Naureckas S, Slater J, Gearhart N, Ramachandran P, Nissinen R, Hopkins L, Geldhof A. Pregnancy outcomes in women with psoriasis and psoriatic arthritis exposed to ustekinumab. Journal of the American Academy of Dermatology 2016;1:AB264. | Conference abstract. Presents data from clinical trials, registries, and spontaneous reports. No usable data. |
| Nissen SE, Wolski K. Effect of Rosiglitazone on the Risk of Myocardial Infarction and Death from Cardiovascular Causes. NEJM. 2007;365:2457-71. | Includes 7 published trials and 35 unpublished trials for AEs of rosiglitazone but does not pool the data for the unpublished and published trials separately. |
| Omboni S, Borghi C. Zofenopril and incidence of cough: a review of published and unpublished data. Therap Clin Risk Manag. 2011;7:459-71. | Did not take the RR/OR against the control arm, but treated as a single arm uncontrolled study where only have estimates of frequency. The method of analysis is inappropriate for pooling risk of AE from RCT data. |
| Palpacuer C, Laviolle B, Boussageon R, Reymann JM, Bellissant E, Naudet F. Risks and Benefits of Nalmefene in the Treatment of Adult Alcohol Dependence: A Systematic Literature Review and Meta-Analysis of Published and Unpublished Double-Blind Randomized Controlled Trials. PLoS Med. 2015;12(12). | Review of the literature including unpublished data. No usable data. |
| Preiss D, Campbel RT, Murray HM, Ford I, Packard CJ, Sattar N, Rahimi K, Colhoun HM, Waters DD, LaRosa JC, Amarenco P, Pedersen TR, Tikkanen MJ, Koren MJ, Poulter NR, Sever PS, Ridker PM, MacFadyen JG, Solomon SD, Davis BR, Simpson LM, Nakamura H, Mizuno K, Marfisi RM, Marchioli R, Tognoni G, Athyros VG, Ray KK, Gotto AM, Clearfield MB, Downs JR, McMurray JJ. The effect of statin therapy on heart failure events: a collaborative meta-analysis of unpublished data from major randomized trials. Eur Heart J 2015;36(24):1536-46. | Review of the literature including unpublished data. No usable data. |
| Psaty BM, Kronmal RA. Reporting Mortality Findings in Trials of Rofecoxib for Alzheimer Disease or Cognitive Impairment. A Case Study Based on Documents From Rofecoxib Litigation. JAMA. 2008;299(15):1813-7. | Numbers of deaths reported in 2 published articles and hazard ratios reported in internal company documents. |
| Ranjan P, Chatterje S, Gupta D, Sardar P, Giri J, Bhinder J, Mukherjee D, Correa A, Kumbhani DJ. Associations of known complications of transcatheter implantation of balloon-expandable edwards-sapien XT aortic valves with peri-procedural mortality-an insight from the 2014-2015 reports from the fda maude database. Circulation. Conference: American Heart Association's 2015;132. | Published adverse effects and case reports from MAUDE database. |
| Rex DK, Deenadayalu VP, Eid E, Imperiale TF, Walker JA, Sandhu K, et al. Endoscopist-Directed Administration of Propofol: A Worldwide Safety Experience. Gastroenterology. 2009;137(4):1229-37. | Did not look at unpublished research studies, but contacted various medical centres to ask them if they had any cases to report. |
| Rising K, Bacchetti P, Bero L. Reporting Bias in Drug Trials Submitted to the Food and Drug Administration : Review of Publication and Presentation. PLoS Medicine. 2008;5(11): e217. | **Comparison of reporting of AE table in published trials and New Drug Applications (NDA) submitted to FDA. P**resence or absence of an AE table reported only not whether there is more or less AE data available. |
| Rosa GM, Ferrero S, Nitti VW, Wagg A; Saleem T; Chapple CR. Cardiovascular Safety of beta3-adrenoceptor Agonists for the Treatment of Patients with Overactive Bladder Syndrome. European Urology 2016;69(2):311-323. | States additional information available from FDA and NICE but no usable data. |
| [Rosenthal R](http://www.ncbi.nlm.nih.gov/pubmed/?term=Rosenthal%20R%5BAuthor%5D&cauthor=true&cauthor_uid=23478520), [Dwan K](http://www.ncbi.nlm.nih.gov/pubmed/?term=Dwan%20K%5BAuthor%5D&cauthor=true&cauthor_uid=23478520). Comparison of randomized controlled trial registry entries and content of reports in surgery journals. [Ann Surg.](http://www.ncbi.nlm.nih.gov/pubmed/23478520) 2013;257(6):1007-15. | Measures discrepancies between trial registry entries and publications. No separate harms data. |
| [Ross JS](http://www.ncbi.nlm.nih.gov/pubmed/?term=Ross%20JS%5BAuthor%5D&cauthor=true&cauthor_uid=19901971), [Mulvey GK](http://www.ncbi.nlm.nih.gov/pubmed/?term=Mulvey%20GK%5BAuthor%5D&cauthor=true&cauthor_uid=19901971), [Hines EM](http://www.ncbi.nlm.nih.gov/pubmed/?term=Hines%20EM%5BAuthor%5D&cauthor=true&cauthor_uid=19901971), [Nissen SE](http://www.ncbi.nlm.nih.gov/pubmed/?term=Nissen%20SE%5BAuthor%5D&cauthor=true&cauthor_uid=19901971), [Krumholz HM](http://www.ncbi.nlm.nih.gov/pubmed/?term=Krumholz%20HM%5BAuthor%5D&cauthor=true&cauthor_uid=19901971). Trial publication after registration in ClinicalTrials.Gov: a cross-sectional analysis. [PLoS Med.](http://www.ncbi.nlm.nih.gov/pubmed/?term=ross+trial+publication+after+registration) 2009 Sep;6(9):e1000144. | **Reports publication rates of records in registry by individual characteristics of studies.** |
| Sharma T, Guski LS, Freund N, Gøtzsche PC. Suicidality and aggression during antidepressant treatment: systematic review and meta-analyses based on clinical study reports. BMJ. 2016;352:i65. | Review of the literature including unpublished data. No usable data. |
| Schwarzer G, Antes G, Tallon D, Egger M. Review publication bias? Matched comparative study of Cochrane and journal meta-analyses. 9th Annual Cochrane Colloquium; 2001Oct 9-13; Lyon, France. 2001 | No harms data. |
| Sharp SC, Hellings JA. (2006). Efficacy and Safety of Selective Serotonin Reuptake Inhibitors in the Treatment of Depression in Children and Adolescents. Clinical Drug Investigation 26(5): 247-255. | No usable data. Searches unpublished and published studies for AEs, but comparative results not presented. |
| [Smith SM](http://www.ncbi.nlm.nih.gov/pubmed/?term=Smith%20SM%5BAuthor%5D&cauthor=true&cauthor_uid=23962590), [Wang AT](http://www.ncbi.nlm.nih.gov/pubmed/?term=Wang%20AT%5BAuthor%5D&cauthor=true&cauthor_uid=23962590), [Pereira A](http://www.ncbi.nlm.nih.gov/pubmed/?term=Pereira%20A%5BAuthor%5D&cauthor=true&cauthor_uid=23962590), [Chang RD](http://www.ncbi.nlm.nih.gov/pubmed/?term=Chang%20RD%5BAuthor%5D&cauthor=true&cauthor_uid=23962590), [McKeown A](http://www.ncbi.nlm.nih.gov/pubmed/?term=McKeown%20A%5BAuthor%5D&cauthor=true&cauthor_uid=23962590), [Greene K](http://www.ncbi.nlm.nih.gov/pubmed/?term=Greene%20K%5BAuthor%5D&cauthor=true&cauthor_uid=23962590), [Rowbotham MC](http://www.ncbi.nlm.nih.gov/pubmed/?term=Rowbotham%20MC%5BAuthor%5D&cauthor=true&cauthor_uid=23962590), [Burke LB](http://www.ncbi.nlm.nih.gov/pubmed/?term=Burke%20LB%5BAuthor%5D&cauthor=true&cauthor_uid=23962590), [Coplan P](http://www.ncbi.nlm.nih.gov/pubmed/?term=Coplan%20P%5BAuthor%5D&cauthor=true&cauthor_uid=23962590), [Gilron I](http://www.ncbi.nlm.nih.gov/pubmed/?term=Gilron%20I%5BAuthor%5D&cauthor=true&cauthor_uid=23962590), [Hertz SH](http://www.ncbi.nlm.nih.gov/pubmed/?term=Hertz%20SH%5BAuthor%5D&cauthor=true&cauthor_uid=23962590), [Katz NP](http://www.ncbi.nlm.nih.gov/pubmed/?term=Katz%20NP%5BAuthor%5D&cauthor=true&cauthor_uid=23962590), [Lin AH](http://www.ncbi.nlm.nih.gov/pubmed/?term=Lin%20AH%5BAuthor%5D&cauthor=true&cauthor_uid=23962590), [McDermott MP](http://www.ncbi.nlm.nih.gov/pubmed/?term=McDermott%20MP%5BAuthor%5D&cauthor=true&cauthor_uid=23962590), [Papadopoulos EJ](http://www.ncbi.nlm.nih.gov/pubmed/?term=Papadopoulos%20EJ%5BAuthor%5D&cauthor=true&cauthor_uid=23962590), [Rappaport BA](http://www.ncbi.nlm.nih.gov/pubmed/?term=Rappaport%20BA%5BAuthor%5D&cauthor=true&cauthor_uid=23962590), [Sweeney M](http://www.ncbi.nlm.nih.gov/pubmed/?term=Sweeney%20M%5BAuthor%5D&cauthor=true&cauthor_uid=23962590), [Turk DC](http://www.ncbi.nlm.nih.gov/pubmed/?term=Turk%20DC%5BAuthor%5D&cauthor=true&cauthor_uid=23962590), [Dworkin RH](http://www.ncbi.nlm.nih.gov/pubmed/?term=Dworkin%20RH%5BAuthor%5D&cauthor=true&cauthor_uid=23962590). Discrepancies between registered and published primary outcome specifications in analgesic trials: ACTTION systematic review and recommendations. [Pain.](http://www.ncbi.nlm.nih.gov/pubmed/?term=discrepancies+between+registered+and+published+smith) 2013;154(12):2769-74 | Measures discrepancies in registry primary outcomes and publication. No separate harms data. |
| Son C; Bartanusz V. Lack of evidence for publication bias in industry funded clinical trials of degenerative diseases of the spine. Journal of Neurosurgery. 2015;123 (2):A521 | Conference abstract only. Presents data on publication rates and industry bias. |
| [Su CX](http://www.ncbi.nlm.nih.gov/pubmed/?term=Su%20CX%5BAuthor%5D&cauthor=true&cauthor_uid=25626862), [Han M](http://www.ncbi.nlm.nih.gov/pubmed/?term=Han%20M%5BAuthor%5D&cauthor=true&cauthor_uid=25626862), [Ren J](http://www.ncbi.nlm.nih.gov/pubmed/?term=Ren%20J%5BAuthor%5D&cauthor=true&cauthor_uid=25626862), [Li WY](http://www.ncbi.nlm.nih.gov/pubmed/?term=Li%20WY%5BAuthor%5D&cauthor=true&cauthor_uid=25626862), [Yue SJ](http://www.ncbi.nlm.nih.gov/pubmed/?term=Yue%20SJ%5BAuthor%5D&cauthor=true&cauthor_uid=25626862), [Hao YF](http://www.ncbi.nlm.nih.gov/pubmed/?term=Hao%20YF%5BAuthor%5D&cauthor=true&cauthor_uid=25626862), [Liu JP](http://www.ncbi.nlm.nih.gov/pubmed/?term=Liu%20JP%5BAuthor%5D&cauthor=true&cauthor_uid=25626862). Empirical evidence for outcome reporting bias in randomized clinical trials of acupuncture: comparison of registered records and subsequent publications. [Trials.](http://www.ncbi.nlm.nih.gov/pubmed/25626862) 2015;16:28. | Compared consistency between registered records and publications. No separate harms data. |
| Szczotka-Flynn L, Diaz M. Risk of corneal inflammatory events with silicone hydrogel and low Dk hydrogel extended contact lens wear: A meta-analysis. Optometry and Vision Science 2007;84(4): 247-256. | Systematic review with no usable data |
| Temple AR, Temple BR, Kuffner EK. Dosing and antipyretic efficacy of oral acetaminophen in children. Clinical Therapeutics. 2013;35(9):1361-75.e1-45. | Did not take the RR/OR against the control arm, but treated as a single arm uncontrolled study where only have estimates of frequency. The method of analysis is inappropriate for pooling risk of AE from RCT data |
| Thakrar DB; Tatt ID; Oestergaard MZ. Extent of publication bias in observational studies investigating adverse drug events. Pharmacoepidemiology and Drug Safety 2015;24:584-585. | Conference abstract only. Tests for publication bias. |
| Udell JA, [Zawi R](http://www.ncbi.nlm.nih.gov/pubmed/?term=Zawi%20R%5BAuthor%5D&cauthor=true&cauthor_uid=24150467), [Bhatt DL](http://www.ncbi.nlm.nih.gov/pubmed/?term=Bhatt%20DL%5BAuthor%5D&cauthor=true&cauthor_uid=24150467), [Keshtkar-Jahromi M](http://www.ncbi.nlm.nih.gov/pubmed/?term=Keshtkar-Jahromi%20M%5BAuthor%5D&cauthor=true&cauthor_uid=24150467), [Gaughran F](http://www.ncbi.nlm.nih.gov/pubmed/?term=Gaughran%20F%5BAuthor%5D&cauthor=true&cauthor_uid=24150467), [Phrommintikul A](http://www.ncbi.nlm.nih.gov/pubmed/?term=Phrommintikul%20A%5BAuthor%5D&cauthor=true&cauthor_uid=24150467), [Ciszewski A](http://www.ncbi.nlm.nih.gov/pubmed/?term=Ciszewski%20A%5BAuthor%5D&cauthor=true&cauthor_uid=24150467), [Vakili H](http://www.ncbi.nlm.nih.gov/pubmed/?term=Vakili%20H%5BAuthor%5D&cauthor=true&cauthor_uid=24150467), [Hoffman EB](http://www.ncbi.nlm.nih.gov/pubmed/?term=Hoffman%20EB%5BAuthor%5D&cauthor=true&cauthor_uid=24150467), [Farkouh ME](http://www.ncbi.nlm.nih.gov/pubmed/?term=Farkouh%20ME%5BAuthor%5D&cauthor=true&cauthor_uid=24150467), [Cannon CP](http://www.ncbi.nlm.nih.gov/pubmed/?term=Cannon%20CP%5BAuthor%5D&cauthor=true&cauthor_uid=24150467). Association between influenza vaccination and cardiovascular outcomes in high-risk patients: a meta-analysis. JAMA 2013;310(16): 1711-20. | Objective was to determine if influenza vaccination is associated with prevention of cardiovascular events. Not a harms analysis. |
| Van Bambeke F, Tulkens PM. Safety profile of the respiratory fluoroquinolone moxifloxacin: comparison with other fluoroquinolones and other antibacterial classes. Drug Safety 2009;32(5): 359-378. | Compares case reports in published literature and from FDA. |
| [Walker KF](http://www.ncbi.nlm.nih.gov/pubmed/?term=Walker%20KF%5BAuthor%5D&cauthor=true&cauthor_uid=25057391), [Stevenson G](http://www.ncbi.nlm.nih.gov/pubmed/?term=Stevenson%20G%5BAuthor%5D&cauthor=true&cauthor_uid=25057391), [Thornton JG](http://www.ncbi.nlm.nih.gov/pubmed/?term=Thornton%20JG%5BAuthor%5D&cauthor=true&cauthor_uid=25057391). Discrepancies between registration and publication of randomised controlled trials: an observational study. [JRSM Open.](http://www.ncbi.nlm.nih.gov/pubmed/25057391) 2014;9;5(5):2042533313517688. | Measures discrepancies in registry information and publication. No separate harms data. |
| Wolfe SM. Selective clinical trial reporting: Betraying trial participants, harming patients. BMJ (Online) 2015;350(h2753). | Editorial. Checked references. |
| [You B](http://www.ncbi.nlm.nih.gov/pubmed/?term=You%20B%5BAuthor%5D&cauthor=true&cauthor_uid=22162583), [Gan HK](http://www.ncbi.nlm.nih.gov/pubmed/?term=Gan%20HK%5BAuthor%5D&cauthor=true&cauthor_uid=22162583), [Pond G](http://www.ncbi.nlm.nih.gov/pubmed/?term=Pond%20G%5BAuthor%5D&cauthor=true&cauthor_uid=22162583),[Chen EX](http://www.ncbi.nlm.nih.gov/pubmed/?term=Chen%20EX%5BAuthor%5D&cauthor=true&cauthor_uid=22162583). Consistency in the analysis and reporting of primary end points in oncology randomized controlled trials from registration to publication: a systematic review. [J Clin Oncol.](http://www.ncbi.nlm.nih.gov/pubmed/22162583) 2012;30(2):210-6 | Measures consistency in primary outcomes in registries and publication. No separate harms data. |
