# Supplementary material for: Reporting of Adverse Events in Published and Unpublished Studies of Health Care Interventions: A Systematic Review
Source: PLoS Med. 2016 Sep 20;13(9):e1002127. doi: 10.1371/journal.pmed.1002127 (PMC5029817; doi:10.1371/journal.pmed.1002127)
Supplement: S3 Table — (DOCX) [file pmed.1002127.s007.docx]

| **Design and risk of bias: Matched cohorts** | | | | | | | | | | |
| --- | --- | --- | --- | --- | --- | --- | --- | --- | --- | --- |
| **Study ID** | **Design** | **Adequacy of search** | **Blinding to publication status** | | **Independent double data extraction** | **Classification of publication status** | | | **External Validity** | **Methods of Matching** |
| Carragee 2011 | Comparison of industry-sponsored publications, subsequent publications and FDA summaries of rhBMP-2 safety. | Identified trials from MEDLINE, reference checking and government and administrative databases (eg, FDA, Centers for Disease Control and Prevention, etc). | NR | | NR | Published trials from literature search on MEDLINE and unpublished trials from FDA data summaries | | | Range of AEs, but only one intervention and one unpublished trial with comparative data presented. | Studies were matched but no details on methods of matching. |
| Hartung 2014 | Discrepancies between ClinicalTrials.gov and publications. | Unpublished studies were identified from CT.gov. If publications not identified using citations within ClinicalTrials.gov, searched MEDLINE and the Cochrane Central Register of Controlled Trials using study interventions, condition, principal investigator and date of trial completion. | NR | | No, one reviewer extracted data and a subsample was independently verified | Published trials from PubMed, MEDLIINE or CENTRAL and unpublished trials from ClinicalTrials.gov | | | Any AEs and any interventions (95% drug, 3% device, 3% other) within ClinicalTrials.gov | Citations provided within ClinicalTrials.gov, or by National Clinical Trial (NCT) identifiers. Considered publication to be a match if the intervention was the same and 1 or more groups in the trial had an identical number of participants. |
| Hodkinson 2016 | Comparison of journal publications and CSRs of orlistat trials | Publications identified from MEDLINE and Cochrane CENTRAL and CSRs from company. | NR | | For three trials one researcher extracted and checked by second researcher. No disagreements therefore final two trials data extracted by one researcher. | Unpublished trials from CSRs and publications from journal articles. | | | Range of AEs, but only one intervention. Five published and unpublished trials. | Company was asked to provide corresponding CSRs for each publication identified. |
| Hughes 2014 | Comparison of reporting in industry sponsored trial registries and publications on antidepressant and antipsychotic drugs | Clinical trials summaries were retrieved from clinicalstudyresults.org. Published journal articles were identified using the bibliography listed in each trial summary. Attempted to manually search PubMed to match possible additional publications with the trial summaries, but the absence of trial identification numbers in journal articles made it extremely difficult to crosscheck and match all sources reliably. These additional efforts, therefore, did not affect the final sample size. | NR | | One coder extracted data and a second and third coder independently extracted these data. The values obtained by the second and third coders were compared to those obtained by the first. Any discrepancies were resolved by consensus. | Unpublished trials from clinicalstudyresults.org and published trials were journal articles identified from bibliography in unpublished trial | | | Two classes of drugs with any AEs. Large number of unpublished and published trials. | Compared trial registry and first associated journal article. No details on methods of matching. |
| Jefferson 2011 | Included CSRs in a systematic review of neuraminidase inhibitors for influenza | CSR reports from industry and corresponding published trials identified as part of a Cochrane review | No | | Yes | Published trials from journal articles and unpublished trials from CSR | | | All AEs for one intervention | No details of methods of matching. |
| Kohler 2015 | Comparison of CSRs of 15 drugs within HTA reports of a single organization (IQWIG) vs journal publications (with CSR used as gold standard) | Relied on search used in AMNOG process (checked by IQWIG) | NR | One author covered all studies and a second author checked extraction and coding. Discrepancies were resolved by consensus. | | | Published sources were journal publications, EPARs and clinicaltrials.gov. Unpublished data were from CSRs and were presented in the HTA report. | 15 drugs, any AEs | | Manufacturers match CSRs and journal publications in the dossier and include full text references, so an extra matching step was not deemed necessary. |
| Le Noury 2015 | Compares data from a trial of paroxetine and imipramine from CSR and a published version | NR | No | NR | | | Published trial from journal article and unpublished trial from corresponding CSR | All AEs for two drug interventions | | Compares one study. No details of methods of matching. |
| Mattila 2011 | **Comparison of EPARs and publications for insomnia medication** | A literature search was performed in PubMed, Embase, JSTOR, NICE, Clinical Evidence, Web of Science, Cochrane and PsycInfo. In addition, references in review articles and identified publications, related articles and lists of publications on company websites were examined. For trials where no publication was found, tried to contact the primary investigator, the sponsor or authors of other publications for the same product. | NR | NR | | | Published trials from PubMed, Embase etc. Only full article publications of trials in peer reviewed journals were considered as publications, thus abstracts or presentations at meetings were not included. Unpublished data from EPARs. | 3 drugs, any AEs | | A trial was included in this study if the EPAR provided sufficient information to identify the matching publication. |
| Maund 2014 | **Comparison of CSRs, trial registries and publications of duloxetine for depression** | CSRs were obtained from the EMA. Journal articles were searched for in PubMed and Cochrane Central Register of Controlled Trials and contacting the manufacturer, Eli Lilly. Clinicaltrials.gov and the manufacturers online clinical trial registry were searched for trial results. | NR | Data from trial registry reports were extracted by one observer and checked by a second. Any discrepancies were resolved by discussion. A third opinion was sought when necessary. | | | Published articles were journal articles identified from database searching and contacting manufacturer. Unpublished trials from CSR and Clinicaltrials.gov. | One drug, any AEs. | | One researcher identified relevant trials based on study ID, indication, sample size, study duration, and dose groups. When there was doubt as to whether a paper should be included, consensus was sought with a second researcher. |
| Pang 2011 | **Comparison conference abstract/journal articles against ‘gold standard’ of 43 phase III/IV trials involving four drugs in the GSK registry** | Examined the GSK register in April 2009 and looked for matched publication in PubMed, and reference list of company trial report | No blinding | Single extractor only | | | Published materials consisted of conference proceedings, abstracts, and journal articles that were accessible electronically. Unpublished trials from GSK Clinical Trial Register | Four newly licensed drugs, any AEs | | Corresponding publications were identified based on citation in reference list, otherwise through matching of drug name, number of participants, and study duration. |
| Pranić 2015 | **Compared trial data from ClinialTrials.gov and publications** | Unpublished studies from Clinicaltrials.gov 2009 to 2012. A search was performed for corresponding published study in MEDLINE with study identifier. A search of Web of Science was also performed with author name and study title. | NR | One investigator extracted data and a second investigator independently reviewed data in a 10% random sample. | | | Published trials from MEDLINE. Unpublished data from ClinicalTrials.Gov | Range of interventions but mostly drug (n=62, 77%). | | Trial study identifier from ClinicalTrials.gov. |
| Riveros 2013 | **Comparison of results in ClinicalTrials.gov and publications** | Searched ClinicalTrials.gov: Of all eligible trials (n = 1,592), a random sample of 600 trials were selected for which to search for full-text publications.  Searched PubMed using the ClinicalTrials.gov identification number (NCT number). If no publication was identified, searched PubMed again using keywords for drug names and the condition studied. | Not blinded because blinding would have been impossible to achieve. | All data were extracted in duplicate by two reviewers. All disagreements were resolved by discussion, including intervention of a third reviewer in case of discrepancies. | | | Published trials were journal articles from link in Clinicaltrials.gov and from PubMed and unpublished trials from Clinicaltrials.gov | Any drug, any AE | | Used the link within ClinicalTrials.gov and ClinicalTrials.gov identification number. Articles identified via PubMed had to match the corresponding trial in terms of the information registered at ClinicalTrials.gov (i.e., same objective, same sample size, same primary outcome, same location, same responsible party, same trial phase, and same sponsor). Second reviewer checked matching between ClinicalTrials.gov and published article. All disagreements were resolved by discussion. |
| Rodgers 2013 | **Comparison of confidential and published data on rhBMP-2 for spinal fusion** | Search of multiple databases with comprehensive search strategy. Unpublished data from manufacturer. | No | One researcher extracted data and a second reviewer checked the data extraction. Resolved discrepancies through consensus or recourse to a third reviewer. | | | Published data included journal articles and conference proceedings. Unpublished data from manufacturer | Any AEs for one intervention | | No details on methods of matching |
| Scharf 2006 | **Comparison of reporting in publications with National Cancer Institute database** | Searched the NCI database. A PubMed search using the agent names and principal investigators. | NR | NR | | | Publications from peer-reviewed journals in PubMed and unpublished data from the Clinical Data Update System (CDUS) of the National Cancer Institute (NCI) | Cancer drug trials only | | The number of patients reported in the articles was confirmed to be identical to the number of patients in CDUS for every trial. |
| Tang 2015 | **Comparison of reporting in ClinicalTrials.gov and journal articles** | Unpublished studies from ClinicalTrials.gov. For publications used link to publications provided by ClinicalTrials.gov and PubMed searched using ClinicalTrials.gov identification number or keywords for drug names and condition studied. | NR | All data were extracted independently by two reviewers. All disagreements were resolved by discussion or a third reviewer if needed. | | | Published trials from journal publication. Unpublished trials from ClinicalTrials.gov | Random sample of all records from ClinicalTrials.gov | | A second reviewer checked the matching and disagreements were resolved by discussion or a third reviewer if needed. |
| Wieseler 2012/2013 | **Comparison of reporting in** registry reports, CSRs, and journal publications | Search generally covered MEDLINE, Embase, and the databases of the Cochrane Library, as well as ClinicalTrials.gov, the International Clinical Trials Registry Platform of the World Health Organization, the Clinical Trials Portal of the International Federation of Pharmaceutical Manufacturers and Associations, the Clinical Study Results Database of the Pharmaceutical Research and Manufacturers of America, and the trial registries and results databases of the manufacturers of the drugs under investigation. In addition, for all HTAs, CSRs were requested from the manufacturers of the drugs under assessment. | NR | All data were extracted and coded by one author. All data from registry reports and all classifications of patient-relevant outcomes were independently checked by a second author. In addition, a random sample of 10% of the data and codings for trial outcomes from CSRs and journal publications was also independently checked by a second author. Discrepancies were resolved by consensus, if necessary, after discussion with a third author. | | | Published trials from journal publication. Unpublished trials from trial registries and CSRs | 16 drug HTAs | | Used ‘paired samples’ |
| **Design and Risk of Bias: Unmatched cohorts** | | | | | | | | | | |
| **Study ID** | **Design** | **Adequacy of search** | **Blinding to publication status** | | **Independent double data extraction** | **Classification of publication status** | | | **External Validity** | **Confounding** |
| Hemminki 1980 | Comparison of proportion of trials that gave information on adverse effects. | All reports of clinical trials on the efficacy of the drugs that were submitted to the Finnish and Swedish drug licensing authorities before the final decision on licensing were included in the analysis. | NR | | NR | Search method unclear; defined as published if found in a journal/ book or meeting report. | | | Wide range of drugs from 191 licensing applications, but these covered the 1960’s and 70’s and may not reflect current practice | Trial quality was checked, but no assessment or adjustment for potential differences. |
| Hughes 2014 | Comparison of reporting in industry sponsored trial registries and publications on antidepressant and antipsychotic drugs | Clinical trials summaries were retrieved from clinicalstudyresults.org. Published journal articles were identified using the bibliography listed in each trial summary. Attempted to manually search PubMed to match possible additional publications with the trial summaries, but the absence of trial identification numbers in journal articles made it extremely difficult to crosscheck and match all sources reliably. These additional efforts, therefore, did not affect the final sample size. | NR | | One coder extracted data and a second and third coder independently extracted these data. The values obtained by the second and third coders were compared to those obtained by the first. Any discrepancies were resolved by consensus. | Unpublished trials from clinicalstudyresults.org and published trials were journal articles identified from bibliography in unpublished trial | | | Two classes of drugs with any AEs. Large number of unpublished and published trials. | Characteristics described but no adjustment for potential differences. |
| Wieseler 2012/2013 | **Comparison of reporting in** registry reports, CSRs, and journal publications | Search generally covered MEDLINE, Embase, and the databases of the Cochrane Library, as well as ClinicalTrials.gov, the International Clinical Trials Registry Platform of the World Health Organization, the Clinical Trials Portal of the International Federation of Pharmaceutical Manufacturers and Associations, the Clinical Study Results Database of the Pharmaceutical Research and Manufacturers of America, and the trial registries and results databases of the manufacturers of the drugs under investigation. In addition, for all HTAs, CSRs were requested from the manufacturers of the drugs under assessment. | NR | | All data were extracted and coded by one author. All data from registry reports and all classifications of patient-relevant outcomes were independently checked by a second author. In addition, a random sample of 10% of the data and codings for trial outcomes from CSRs and journal publications was also independently checked by a second author. Discrepancies were resolved by consensus, if necessary, after discussion with a third author. | Published trials from journal publication. Unpublished trials from trial registries and CSRs | | | 16 drug HTAs | Some characteristics described but no adjustment for potential differences. |
| Connolly 2013 | Comparison of data retrieved from investigators with published trials on aspirin therapy and risk of subdural hematoma. | Searched Cochrane Stroke group register and checked references. Contacted investigators and requested unpublished data. | NR | | Two reviewers independently extracted data. | Published trials from literature search for subset of CENTRAL and unpublished trials from contacting investigators. | | | One AE with one drug intervention. | Summaries characteristics of published trials and unpublished trials. Participants in unpublished trials more likely to be younger and female and use agents for primary prevention. Unpublished trials more likely to have a larger number of participants, to have longer follow-up and higher Jadad score. |
| Eyding 2010 | Comparison of published and unpublished trials of reboxetine or SSRIs for depression. | Searched Medline, Embase, PsycINFO, BIOSIS, and the Cochrane Library, checked reference lists, screened clinical trial registries and trial results databases and websites of the European Medicines Agency (EMA) and US Food and Drug Administration (FDA).Unpublished data and trials from manufacturer. | NR | | Data were extracted by one reviewer and checked by another. | Published trials from literature search and unpublished trial data from manufacturer | | | Patients with AEs, SAEs, withdrawals to AEs; all studies directly comparing one drug against placebo or other antidepressants identified by a systematic search Limited number of published trials. | No mention of confounders. |
| Hart 2012 | Comparison of results of meta-analysis with and without FDA unpublished trial data for Pimecrolimus | Searched Cochrane Skin Group Specialised Register, CENTRAL, Medline, Embase and checked references. Contacted researchers and manufacturers. Unpublished and ongoing trials by checking following websites - European Agency for the Evaluation of Medicinal Products website, US Food and Drug administration , manufacturer of pimecrolimus (Novartis) clinical trial results, meta Register of Current Controlled trials, and Cochrane Skin Group Ongoing Skin Trials Register | NR | | Two authors independently extracted data. | Unpublished data from FDA trials | | | Any AEs with one drug. Unknown number of published and unpublished trials. | No mention of confounders. |
| Hemminki and McPherson 2000 | Cardiovascular and thrombotic events with postmenopausal hormone therapy with meta-analysis of published trials vs unpublished data. | Used clinical trials included in applications submitted to licensing authorities in Finland and Sweden. | NR | | Only the author extracted data | No definitions of published and unpublished studies; authors acknowledge potential misclassification | | | Limited to a single drug class. | No mention of confounders. |
| MacLean 2003 | **Meta-analysis of dyspepsia with NSAIDs** | Searched Medline, Embase, HealthStar and BIOSIS and FDA reports. | NR | | One reviewer extracted data. 10% sample extracted by two reviewers to determine reliability. | ‘Unpublished’ trials from FDA reviews, published ones retrieved from electronic databases; trial characteristics were cross-checked. | | | Limited to NSAIDs only. | Potential confounders looked at, and meta-regression used to assess differences. |
| Moja 2014 | **SR on safety of** bevacizumab versus ranibizumab for neovascular age-related macular degeneration | Searched CENTRAL, MEDLINE, Emabse, clinical trial registries, metaRegister of Controlled Trials, clinicaltrials.gov, WHO International Clinical Trials Registry Platform (ICTRP) and reference lists. For unpublished trials searched the Internet for conferences/meeting and contacted authors. | NR | | Two investigators independently extracted data on  study characteristics. Three investigators extracted data on our safety primary  and secondary outcomes . | Unpublished data from pre-publication study presentations at conferences or meetings and study authors. | | | Schwarz review on two drugs for one condition. | No mention of confounders. |
| Potthast 2014 | **Compares SR results with and without data from industry trial registries** | Results registries searched by Potthast et al for each review. Schwarz review originally searched Cochrane Schizophrenia Group’s Register and checked references. For unpublished trials contacted manufacturer and authors of included studies.  Cipriani review originally searched Depression, Anxiety and Neurosis Group register, CENTRAL, MEDLINE, Embase, Cinahl, PsycINFO and carried out handsearching, reference checking as well as contacting authors and manufacturers. | NR | | One author from Potthast et al carried out screening and analysis and this was checked by a second author. In the Schwarz review independent data extraction and disagreements discussed. In the Cipriani review 2 authors independently carried out data extraction and disagreements resolved by discussion with third author. | If results registries not searched in SR Potthast et al searched for unpublished data from industry results registries and clinicalstudyresults.org and ifpma.org. | | | Schwarz review on valproate for schizophrenia and Cipriani review on olanzapine for bipolar disorder. | No mention of confounders by Potthast 2014. |
| Ross 1997 | **Meta-analysis of serious adverse events in hypertensive patients receiving isradipine.** | Searched MEDLARS, Current Contents, reference checking, and contacted manufacturers. | NR | | Data extraction by at least two reviewers. | ‘Unpublished’ trials from company reports, published ones retrieved from electronic databases; trial characteristics were cross-checked. | | | Limited to isradipine only. | Unpublished trials were all from industry, and trial quality may differ, |
| Singh-Franco 2012 | **Meta-analysis of linagliptin tolerability in patients with type 2 diabetes.** | Searched Medline, Embase CENTRAL, Cochrane Methodology Register, Cochrane Database of Systematic Reviews, Database of Abstracts of Reviews of Effects, Biomedical Reference Collection, CINAHL, Nursing and Allied Health Collection and Ageline. Checked references. Handsearched journals and used own files. Authors and manufacturers were contacted for data. | NR | | NR | Published trials from literature searches. Unpublished trials from clinicaltrials.gov. | | | One drug, any AEs | No mention of confounders |
| Wallace 2006 | **Meta-analysis of serious adverse events with SSRIs** | Searched Medline, Cinahl, Biosis, and CENTRAL. Checked references and handsearched journals. Unpublished trials from the Committee on Safety of Medicines (CSM) website. | NR | | Two authors independently extracted data. | ‘Unpublished’ data from UK regulatory agency, not clear how authors established such data was genuinely unpublished | | | Limited to SSRIs only. | Study characteristics were described, but no discussion of adjustment for differences between published and unpublished |
| Whittington 2004 | **Meta-analysis of** serious adverse events attributable to **SSRIs.** | Searched Embase, Medline, PsycINFO, CINAHL, and the Cochrane Library. Checked references, handsearched journals, contacting experts. Unpublished data from CSM reports. | NR | | One reviewer extracted data. Double-checked by another author. | ‘Unpublished data’ from UK regulatory agency, not clear how authors established such data were genuinely unpublished | | | Limited to SSRIs only. | Study characteristics were described, but no discussion of adjustment for differences between published and unpublished |
