# Supplementary material for: Reporting of Adverse Events in Published and Unpublished Studies of Health Care Interventions: A Systematic Review
Source: PLoS Med. 2016 Sep 20;13(9):e1002127. doi: 10.1371/journal.pmed.1002127 (PMC5029817; doi:10.1371/journal.pmed.1002127)
Supplement: S4 Table — (DOCX) [file pmed.1002127.s008.docx]

| **Reference** | **Main Outcome Measures and Results** |
| --- | --- |
| **1a. Compares number of studies reporting AEs information in matched published and unpublished articles** | |
| Hughes 2014 | **Number of studies that report number of SAEs**  85/142 (60%) published trials vs 125/142 (88%) associated registry reports vs (95/102 (93%) unrelated registry reports) |
| Kohler 2015 | **Rates of complete information in HTA reports of which 42 publications from 21 matched studies available**  **Any AEs**  Total study population: Publications 6/15 (40%) vs 14/15 (93%) AMNOG  Subpopulation of approved indication: Publications 0/13 (0%) vs 11/13 (85%) AMNOG  **Any SAEs**  Total study population: Publications 5/15 (33%) vs 14/15 (93%) AMNOG  Subpopulation of approved indication: Publications 0/13 (0%) vs 10/13 (77%) AMNOG  **Any Withdrawals due to AEs**  Total study population: Publications 7/15 (47%) vs 14/15 (93%) AMNOG  Subpopulation of approved indication: Publications 0/13 (0%) vs 10/15 (77%) AMNOG |
| Mattila 2011 | Safety information missing in 8/15 (53%) publications compared to unpublished trials from EPARs, with one publication lacking any safety data. |
| Maund 2014 | **Reporting in full for 9 trials**  **Death reported in full**  Published trials 3/9 (33%) vs CSRs 9/9 (100%) vs registry reports 6/9 (67%)  **SAEs reported in full (number of patients)**  Published trials 3/9 (33%) vs CSRs 9/9 (100%) vs registry reports 3/9 (33%)  **SAEs reported in full (number of events)**  Published trials 3/9 (33%) vs CSRs 9/9 (100%) vs registry reports 3/9 (33%)  **Withdrawals due to AEs reported in full (number of patients)**  Published trials 7/9 (78%) vs CSRs 9/9 (100%) vs registry reports 7/9 (78%)  **Withdrawals due to AEs reported in full (number of events)**  Published trials 1/9 (11%) vs CSRs 9/9 (100%) vs registry reports 1/9 (11%)  **Treatment emergent AEs reported in full (number of patients)**  Published trials 0/9 (0%) vs CSRs 9/9 vs registry reports 9/9 (100%)  **Treatment emergent AEs (number of events)**  Published trials 0/9 (0%) vs CSRs 9/9 (100%) vs registry reports 0/9 (0%)  **Discontinuation emergent AEs reported in full (number of patients)**  Published trials 0/9 (0%) vs CSRs 9/9 (100%) vs registry reports 0/9 (0%)  **Discontinuation emergent AEs reported in full (number of events)**  Published trials 0/9 (0%) vs CSRs 9/9 (100%) vs registry reports 0/9 (0%)  **Suicide reported in full**  Published trials 2/2 (100%) vs CSRs 2/2 (100%) vs registry reports 2/2 (100%)  **Attempted suicide reported in full (number of patients)**  Published trial 0/1 (0%) vs CSR 1/1 (100%) vs registry report 0/1 (0%)  **Attempted suicide reported in full (number of events)**  Published trial 0/1 (0%) vs CSR 1/1 (100%) vs registry report 0/1 (0%) |
| Pranić 2015 | **Number of trials reporting SAEs (“81 trials with 21 corresponding publications”)**  Published trials 7/21 (33%) vs unpublished trials from trial registry 38/81 (47%)  **Number of trials reporting other AEs**  Published trials 15/21 (71%) vs unpublished trials from trial registry 55/81 (68%) |
| Riveros 2013 | **Number of trials reporting total number of AEs**  Published trials 128/202 (63%) vs registry reports 194/202 (96%)  **Number of trials reporting details of all AEs per arm**  Published trials 128/202 (63%) vs registry reports 194/202 (96%)  **Number of trials reporting number of withdrawals due to AEs**  Published trials 153/202 (76%) vs registry reports 161/202 (80%)  **Number of trials reporting total number of SAEs**  Publications 144/202 (71%) vs registry reports 200/202 (99%)  **Number of trials reporting details of all SAEs per arm**  Publications 127/202 (63%) vs registry reports 199/202 (99%)  **Number of trials with complete reporting of AEs**  Publications 91/202 (45%) vs registry reports 147/202 (73%)  **AEs**  More information in ClinicalTrials.gov than published article 80/202  Similar information in ClinicalTrials.gov and published article 98/202  More information in publication than ClinicalTrials.gov 24/202  **SAEs**  More information in ClinicalTrials.gov than published article 73/202  Similar information in ClinicalTrials.gov and published article 128/202  More information in publication than ClinicalTrials.gov 1/202 |
| Tang 2015 | **Number of trials reporting SAEs per treatment group**  Publications 139/202 (69%) vs registry reports 202/202 (100%) |
| Wieseler 2012/2013 | **Information provided in registry reports vs journal publications (matched sample of 47 studies)**  **AEs (number of patients)**  More information in registry report than publication 23/47 (49%)  Similar information in registry report and publication 16/47 (34%)  Less information in registry report than publication 8/47 (17%)  **SAEs (number of patients)**  More information in registry report than publication 18/47 (38%)  Similar information in registry report and publication 22/47 (47%)  Less information in registry report than publication 7/47 (15%)  **Withdrawals due to AEs**  More information in registry report than publication 5/47 (10%)  Similar information in registry report and publication 32/47 (68%)  Less information in registry report than publication 10/47 (21%)  **Information provided in registry reports vs CSRs (matched sample of 50 studies)**  **AEs (number of patients)**  More information in registry report than CSR 0/50 (0%)  Similar information in registry report and CSR 41/50 (82%)  Less information in registry report than CSR 9/50 (18%)  **SAEs (number of patients)**  More information in registry report than CSR 1/50 (2%)  Similar information in registry report and CSR 38/50 (76%)  Less information in registry report than CSR 11/50 (22%)  **Withdrawals due to AEs**  More information in registry report than CSR 0/50 (0%)  Similar information in registry report and CSR 43/50 (86%)  Less information in registry report than CSR 7/50 (14%)  **Completeness of information in CSRs vs journal publications (matched sample of CSRs and journal publication N = 65)**  Full information on patients with AEs: CSR: 61/65 (94 %) vs publication: 21/65 (32%)  Full information on patients with SAEs: CSR: 60/65 (92%) vs publication: 24/65 (37%)  Full information on patients withdrawn due to AEs: CSR 61/65 (94%) vs publication 51/65 (78%)  Full information on patients with specific AEs: CSR 183/209 (88%) vs publication 66/209 (32%)  **Completeness of information in CSRs vs registry reports (matched sample of CSRs and registry reports N = 50)**  Full information on patients with AEs: CSRs: 50/50 (100 %) vs registry reports: 41/50 (82%)  Full information on patients with SAEs: CSR: 43/50 (86%) vs registry reports 37/50 (74%)  Full information on patients withdrawn due to AEs: CSR 49/50 (98%) vs registry reports 42/50 (84%)  Full information on patients with specific AEs: CSR 129/155 (83%) vs registry reports 34/155 (22%) |
| **1b. Compares number of studies reporting AEs information in unmatched published and unpublished articles** | |
| Hemminki 1980 | **% of controlled trials giving information on adverse effects;**  Published vs unpublished applications for licensing  *Finland*:  Psychotrophic drugs 56% vs 77%  Non Psychotrophics 43% vs 83%  *Sweden*:  Psychotrophic drugs 73% vs 83% |
| Wieseler 2012/2013 | **Studies with complete information (identified from 16 HTAs)**  **AEs**  65/192 (34%) publications vs 93/101 (92%) CSR vs 57/78 (73%) registry reports  **SAEs**  67/192 (35%) publications vs 89/101 (88%) CSR vs 57/78 (73%) registry reports  **Withdrawals due to AEs**  144/192 (75%) publications vs 92/101 (91%) CSR vs 57/78 (73%) registry reports  **Outcomes with complete information (only trials with full CSR available from 16 HTAs)**  **AEs**  21/101 (21%) in 65 publications vs 93/101 (92%) in 101 CSRs vs 41/101 (41%) in 50 registry reports  **SAEs**  24/101 (24%) in 65 publications vs 89/101 (88%) in 101 CSRs vs37/101 (37%) in 50 registry reports  **Withdrawals due to AEs**  51/101 (50%) in 65 publications vs 92/101 (91%) in 101 CSRs vs 42/101 (42%) in 50 registry reports  **Special AEs**  66/321 (21%) in 65 publications vs 271/321 (84%) in 101 CSRs vs34/321 (11%) in 50 registry reports |
| **2a. Compares numbers of AEs reported in matched published and unpublished studies** | |
| Carragee 2011 | **Number of early infections (number of rhBMP-2 patients, n=277)**  3 published trials 0 vs 1 FDA data summary 26  **Number of delayed infection (n=277)**  3 published trials 0 vs 1 FDA data summary 12  **Number of implant malposition, displacement and loosening (n=277)**  3 published trials 9 vs 1 FDA data summary 10  **Number of subsidence (n=277)**  3 published trials 7 vs 1 FDA data summary 7  **Number of reoperation for device-related AE (n=277)**  3 published trials 7 vs 1 FDA data summary 22  **Number of retrograde ejaculation (n=277)**  3 published trials 0 vs 1 FDA data summary 12  **Number of other urogenital AE (n=277)**  3 published trials 0 vs 1 FDA data summary 36 |
| Hartung 2014 | **Number of SAEs**  38 published trials 4784 vs 38 registry reports 9366 |
| Hodkinson 2016 | **Number of AEs**  5 published trials 42 vs 5 CSRs 118  **Number of SAEs**  5 published trials 15 vs 5 CSRs 375 |
| Hughes 2014 | **Number of SAEs in drug-treated participants**  142 published trials 914 vs 142 registry reports 1,608  **Number of SAEs per patient treated**  142 published trials 0.03 vs 142 registry reports 0.05  **Number of deaths in drug-treated participants**  142 published trials 57 vs 142 registry reports 151  **Number of completed suicides in drug-treated participants**  142 published trials 7 vs 142 registry reports 15  **Number of suicidal ideations, attempts or injury in drug-treated participants**  142 published trials 47 vs 142 registry reports 87  **Number of homicidal ideations in drug-treated participants**  142 published trials 1 vs 142 registry reports 1  **Number of new or worsened psychiatric symptoms in drug-treated participants**  142 published trials 115 vs 142 registry reports 238 |
| Jefferson 2011 | **Number of SAEs**  2 published trials 0 vs 2 CSRs 10 (9 subjects) |
| Le Noury 2015 | **Paroxetine**  **Number of all AEs**  1 published trial 265 vs 1 CSR 338 (vs RIAT 481)  **Number of cardiovascular AEs**  1 published trial 5 vs 1 CSR 7 (vs RIAT 44)  **Number of gastrointestinal/digestive AEs**  1 published trial 84 vs 1 CSR 80 (vs RIAT 112)  **Number of respiratory AEs**  1 published trial 33 vs 1 CSR 39 (vs RIAT 42)  **Number of neurological/nervous system**  1 published trial 115 vs 1 CSR 106 (vs RIAT 101)  **Number of other AEs**  1 published trial 28 vs 1 CSR 121 (vs RIAT 79)  **Number of suicidal and self injurious behaviours**  1 published trial 5 vs 1 CSR 7 (vs RIAT 11)  **Imipramine**  **Number of all AEs**  1 published trial 340 vs 1 CSR 493 (vs RIAT 552)  **Number of cardiovascular AEs**  1 published trial 42 vs 1 CSR 60 (vs RIAT 130)  **Number of gastrointestinal/digestive**  1 published trial 106 vs 1 CSR 108 (vs RIAT 147)  **Number of respiratory AEs**  1 published trial 27 vs 1 CSR 32 (vs RIAT 22)  **Number of neurological/nervous system**  1 published trial 135 vs 1 CSR 117 vs (RIAT 114)  **Number of other AEs**  1 published trial 30 vs 1 CSR 51 vs RIAT 76  **Number of suicidal and self injurious behaviours**  1 published trial 3 vs 1 CSR 3 (vs RIAT 4) |
| Maund 2014 | **Number of treatment emergent AEs**  9 published trials 1558 vs 9 CSRs 6087 vs 9 registry reports 4629 |
| Pranić 2015 | **Number of SAEs (21 publications)**  Higher number and /or frequency in publication 0 (0%)  Higher number and /or frequency in registry reports 1 (5%)  **Number of other AEs (21 publications)**  Higher number and /or frequency in publication 11 (52%)  Higher number and /or frequency in registry reports 2 (10%) |
| Rodgers 2013 | **Number of AEs**  Publications 533 vs data from manufacturer 2302 |
| Scharf 2006 | **Number of AEs**  AEs =>grade 3  22 publications 413 vs Clinical Data Update System (CDUS) 423  **Number of AEs grade 1 to 5 (all AEs)**  22 publications 1910 vs Clinical Data Update System (CDUS) 2995 |
| Tang 2015 | **Number of SAEs**  Number of SAEs greater in registry reports than published article 31  Matching number of SAEs in published and registry reports 95  Number of SAEs greater in published than registry reports 13 |
| **2b. Compares different types of AEs in matched published and unpublished trials** | |
| Pang 2011 | **Description of specific types of ‘Fatal’ AEs**  198 specific fatal events described in GSK trial register; 29 (14.6%) were reported in published version  **Description of specific types of SAEs**  1147 specific types of serious events described in GSK trial register, 76 (6.7%) were reported in published version  **Description of specific types of ‘most frequent’ adverse events**  281 types of frequent events described in GSK trial register, 91(32.4%) were reported in published version  **Total number of types of AEs reported**  1626 types of adverse events described in GSK trial register, 196 (12.1%) were reported in published version |
| Kohler 2015 | **Description of specific AE** (54 different types in CSR for total study population, and 52 different types for subpopulation)  Total study population: 18 (33%) types of AE described in publications vs 49 (91%) types of AE in AMNOG  **Subpopulation with approved indication:** 0 (0%) types of AE described in publications vs 29 (56%) types of AE in AMNOG |
| **2c. Compares numbers of AEs reported in unmatched published and unpublished studies** | |
| Hughes 2014 | **Number of SAEs in drug-treated participants**  142 published trials 914 vs 102 registry reports 1423  **Number of SAEs per patient treated**  142 published trials 0.03 vs 102 registry reports 0.07  **Number of deaths in drug-treated participants**  142 published trials 57 vs 102 registry reports 45  **Number of completed suicides in drug-treated participants**  142 published trials 7 5 vs 102 registry reports 5  **Number of suicidal ideations, attempts or injury in drug-treated participants**  142 published trials 47 vs 102 registry reports 79  **Number of homicidal ideations in drug-treated participants**  142 published trials 1 vs 102 registry reports 5  **Number of new or worsened psychiatric symptoms in drug-treated participants**  142 published trials 115 vs 102 registry reports 357 |
| **3. Compares pooled effect estimates in published and unpublished studies** | |
| Connolly 2013 | **Subdural hematoma**  4 published trials (N=6565) OR 2.2 (0.6-7.8) vs 5 unpublished trials (from contacting investigators) (N=90689) OR 1.3 (0.5-3.5) vs 9 published and unpublished trials (N=97254) OR 1.6 (0.8-3.5) |
| Eyding 2010 | **Reboxetine vs placebo**  **AEs**  2 published trials (N=310) OR 2.67 (0.52-13.79) vs 6 unpublished trials (from manufacturer) (N=1938) OR 2.15 (1.66-2.80) vs 8 published and unpublished trials (N=2248) OR 2.14 (1.59-2.88)  **Withdrawals due to AEs**  2 published trials (N=310) OR 0.95 (0.45-1.99) vs 6 unpublished trials (from manufacturer) (N=1938) OR 2.61 (1.79-3.80) vs 7 published and unpublished (N=2248)2.21 (95% CI 1.45–3.37)  **Reboxetine vs SSRIs**  **AES**  2 published trials (N=421) OR 1.07 (0.72-1.61) vs 5 unpublished trials (from manufacturer) (N=1752) OR 1.08 (0.74-1.58) vs 7 published and unpublished trials (N=2173) OR 1.06 (0.82-1.36)  **Withdrawals due to AEs**  2 published trials (N=421) OR 1.58 (0.81-3.08) vs 2 unpublished trials (from manufacturer) (N=385) OR 1.72 (0.46-6.42) vs 4 published and unpublished (N=806) 1.79 (95% CI 1.06–3.05) |
| Hart 2012 | **Any AEs**  Published trials RR 0.85 (0.71-1.03) vs unpublished trials (from FDA) RR 0.96 (0.84-1.1) vs 4 published and unpublished trials (N=827) RR 0.92 (0.82-1.02) |
| Hemminki and McPherson 2000 | **Cardiovascular events**  22 published trials OR 1.39 (0.48-3.95) vs 28 unpublished and published trials OR 1.78 (0.70-4.52)  **Cardiovascular and thrombotic events**  22 published trials OR 1.64 (0.65-4.21) vs 28 unpublished and published trials OR 1.97 (0.84-4.58) |
| MacLean et al 2003 | **Dyspepsia**  15 published trials (N=1455), RR 1.21; (0.81 – 1.81) vs 11 unpublished trials (from FDA) (N=2368), RR 1.07; (0.70-1.63) vs 26 published and unpublished trials (N=3823) RR 1.14 (0.85-1.53) |
| Moja 2014 | **SAE***  6 published trials 1.21 (1.06-1.37) vs 3 unpublished trials 0.94 (0.58 – 1.50) vs 9 published and unpublished trials (from authors and internet) (N=3665) 1.08 (0.90-1.31)  **Death***  6 published trials 1.12 (0.78-1.62) vs 2 unpublished trials 0.85 (0.22 – 3.38) vs 8 published and unpublished trials (from authors and internet) (N=3338) 1.10 (0.78-1.57) |
| Potthast 2014 | **AE (Schwarz 2009)**  1 published trial (N=249) RR 1.11 (0.98-1.26) vs 1 unpublished trial (from registries) OR 1.6 (0.8-3.5) vs 2 published and unpublished trials RR 1.08 (1.00-1.16)  **Withdrawals due to AE (Cipriani 2009)**  1 published trial (N=361) RR 21.22 (1.29-349.99) vs unpublished data (from registries) RR 1.55 (0.82-2.92) vs published and unpublished data RR 1.76 (0.95-3.28)  There were also three systematic reviews where unpublished additional trial data were available; inclusion of unpublished data did not change significance of results in these instances; but the unpublished data led to additional trials/data for a new comparison not reported in the systematic reviews (Nakagawa 2009 and Cipriani 2009_sertraline) or to additional safety data for a comparison already reported in the systematic review (Ara 2008) |
| Ross 1997 | **Major adverse events in trials**  2 published trials (N=414) OR 0.92 (0.49-1.72) vs 8 unpublished trials (from manufacturer) (N=1988) OR 1.04 (0.64-1.71) vs 10 published and unpublished trials (N=2402) OR 0.99 (0.67-1.46)  **Angina**  2 published trials (from manufacturer) (N=414) OR 0.92 (0.49-1.72) vs 8 unpublished (N=1105) OR 0.99 (0.50-1.97) vs 10 published and unpublished trials (N=1519) OR 1.08 (0.42-2.73) |
| Singh-Franco 2012 | **Any AEs**  5 published trials (N=2784) RR 1.00 (0.91-1.10) vs 4 unpublished trials (from registries) (N=1106) RR 0.84 (0.39-1.81)  **Withdrawals**  5 published trials (N=2784) RR 0.89 (0.50-1.59) vs 4 unpublished trials (from registries) (N=1106) RR 0.58 (0.29-1.18)  **SAEs**  5 published trials (N=2784) RR 0.96 (0.60-1.53) vs 3 unpublished trials (from registries) (N=984) RR 2.85 (0.86-9.43)  **Medication-related AEs**  4 published trials (N=2647) RR 1.24 (0.66-2.32) vs 2 unpublished trials (from registries) (N=336) RR 0.70 (0.39-1.28)  **Hyperglycaemia**  3 published trials (N=2258) RR 0.44 (0.30-0.64) vs 4 unpublished trials (from registries) (N=1106) RR 0.39 (0.19-0.78) vs 7 published and unpublished (N=3364) 0.42 (95% CI 0.31–0.58)  **Musculoskeletal**  4 published trials (N=2395) RR 1.32 (0.98-1.78) vs 2 unpublished trials (from registries) (N=739) RR 2.33 (0.69-7.84) vs 6 published and unpublished (N=3134) RR 1.38 (95% CI 1.03–1.84)  **Gastrointestinal**  3 published trials (N=2281) RR 0.76 (0.58-1.00) vs 3 unpublished trials (from registries) (N=861) RR 2.02 (1.15-3.54)  **Central nervous system**  3 published trials (N=2258) RR 0.90 (0.63-1.28) vs None provided  **Respiratory**  4 published trials (N=2395) RR 0.87 (0.71-1.08) vs 3 unpublished trials (from registries) (N=861) RR 2.22 (0.61-8.03)  **Renal**  2 published trials (N=1755) RR 0.67 (0.40-1.11) vs 2 unpublished trials (from registries) (N=739) RR 0.75 (0.33-1.66)  **Vascular**  4 published trials (N=2647) RR 1.72 (0.98-3.05) vs 1 unpublished trial (from registries) (N=500) RR 0.59 (0.20-1.74) |
| Wallace 2006 | **SAEs for SSRIs**  7 published trials (N=1303); Treated 56/657 vs Placebo 28/646, RR 2.0 (1.3-3.0) vs 11 published and unpublished trials (N=2145) RR 1.97 (1.42-2.75) |
| Whittington 2004 | **SAEs**  1 published trial (N=180); Treated 11/93 - Placebo 2/87, RR 5.15 (1.17-22.56), vs 1 unpublished trial (from UK regulatory agency) (N=275); Treated; 22/182 - Placebo 6/93 RR 1.87 (0.79-4.46) vs 2 published and unpublished trials RR 2.55 (1.23-5.3)  **Suicide attempt or ideation**  1 published trial (N=180); Treated 5/93 - Placebo 0/87, RR 10.30 (0.58 – 183.53) vs published and unpublished from UK regulatory agency(N=663), RR 1.51 (0.62 -3.69) |

*calculated values using the same meta-analytic approach as described by the authors
