# Supplementary material for: Reporting of Adverse Events in Published and Unpublished Studies of Health Care Interventions: A Systematic Review
Source: PLoS Med. 2016 Sep 20;13(9):e1002127. doi: 10.1371/journal.pmed.1002127 (PMC5029817; doi:10.1371/journal.pmed.1002127)
Supplement: S2 Text — (DOCX) [file pmed.1002127.s010.docx]

## Systematic review of the impact of publication status on the reporting of adverse effects

### Objective

To systematically review the literature that has analyzed published versus unpublished information on adverse effects.

## Background

Adverse effects are defined as harmful or undesirable outcomes that occur during or after the use of a drug or intervention for which there is at least a reasonable possibility of a causal relation [1]. Findings from the literature indicate that information on adverse drug effects can be retrieved from unpublished data and industry data that is not available in the published literature. In 2010, Golder and colleagues found that risk estimates of adverse drug effects derived from meta-analyses of unpublished data and meta-analyses of published data do not differ [2]. In addition, risk estimates derived from meta-analyses of industry data and meta-analyses of non-industry data also do not differ [3]. The searches for these studies, however, were carried out in 2009 and no studies were identified which compared published and unpublished data for non-pharmacological interventions.

The importance of publication bias for adverse effects of non-drug interventions and the potential for bias from medical device manufacturer studies is unknown. Unpublished adverse effects data of non-drug interventions may differ from unpublished adverse effects data of drugs and manufacturers of medical devices have different publication patterns for AE data compared to the pharmaceutical industry. Any differences may be due to the regulatory requirements for research evidence on the safety of new devices being universally less stringent than those for medicines, or to more clinicians conducting research into non-drug interventions (such as surgeons investigating the safety of a surgical procedure). The subject of unpublished data has received much attention in recent years due to campaigns such as Alltrialdata, the release of the results in trial registries, and access to clinical study reports.

This systematic review will update the review by Golder et al 2010 [2] to identify more recent comparisons of unpublished and published pharmaceutical data and to identify any comparisons of unpublished and published non-pharmaceutical data.

## Methods

#### Inclusion criteria

Any type of evaluation will be considered eligible for inclusion in this review if it has compared adverse effects of healthcare interventions according to publication status (i.e. published versus unpublished literature). All healthcare interventions are eligible (such as drug interventions, surgical procedures, medical devices, dentistry, screening and diagnostic tests). Eligible articles will be those that reviewed one or more published and unpublished studies and compared the quantitative reporting of adverse effects, in particular, the type and range of adverse effects and the frequency, rate or risk of adverse effects. ‘Published’ articles are generally considered to be manuscripts that are found in peer-reviewed journals. Whereas ‘unpublished’ data can be located through any other avenue (for example, from regulatory websites, industry, personal contact or from conference proceedings).

#### Exclusion criteria

We are primarily concerned with the properties of interventions under normal use. We will therefore not consider the broader range of events such as intentional and accidental poisoning (i.e. overdose), drug abuse, errors, or non-compliance.

#### Search methods

The sources to be searched are listed below;

###### Databases

CINAHL Plus

Cochrane Database of Systematic Reviews (CDSR) – methodology reviews only

Cochrane Methodology Register (CMR)

Conference Proceedings Citation Index (CPCI)

Embase

Health Management Information Consortium (HMIC)

MEDLINE

MEDLINE In Process

OpenGrey

Proquest Dissertations & Theses: UK & Ireland

Proquest Library Science and Library and Information Science & Technology Abstracts (LISTA)

PsycINFO

Science Citation Index (SCI)

Scopus

Zetoc

###### Internet Search Engines

Google

Google Scholar

###### Handsearching of Journals

Drug Safety

Pharmacoepidemiology and Drug Safety

###### Hansearching of Bibliographies

Arber M, Cikalo M, Glanville J, Lefebvre C, Varley D, Wood H. Annotated bibliography of published studies addressing searching for unpublished studies and obtaining access to unpublished data. York: York Health Economics Consortium; 2013.

###### Handsearching of Conference Proceedings

Cochrane Colloquium

Other methods for identifying relevant articles will include, reference checking of all included articles and any related systematic reviews and contacting authors and experts in the field.

#### Search Strategies

The search strategies will contain just two facets – publication status and adverse effects. No population or comparator or study design terms will be incorporated into the search strategy as there are no restrictions on these elements in the inclusion criteria. A date restriction of 2009 onwards will be placed on the searches as this is the date of the last searches carried out. No language restrictions will be placed on the searches, although financial and logistical restraints may not enable the translation from all languages. The MEDLINE search strategy is contained below and will be translated to as appropriate for the search interfaces used with each individual database.

MEDLINE Search strategy

Database: Ovid MEDLINE(R) In-Process & Other Non-Indexed Citations and Ovid MEDLINE(R) <1946 to Present>
Search Strategy:
--------------------------------------------------------------------------------
1     unpublished.ti. (629)
2     "not published".ti. (8)
3     grey literature.ti. (27)
4     gray literature.ti. (6)
5     clinical study report*.ti,ab,kw. (135)
6     regulatory website*.ti,ab,kw. (8)
7     regulatory web site*.ti,ab,kw. (2)
8     regulatory report*.ti,ab,kw. (39)
9     regulatory data.ti,ab,kw. (89)
10     regulatory document*.ti,ab,kw. (138)
11     (fda adj3 document*).ti,ab,kw. (126)
12     (fda adj3 reports).ti,ab,kw. (164)
13     (fda adj3 data).ti,ab,kw. (334)
14     (fda adj3 website).ti,ab,kw. (66)
15     (fda adj3 web site).ti,ab,kw. (45)
16     ("food and drug administration" adj3 document*).ti,ab,kw. (58)
17     ("food and drug administration" adj3 reports).ti,ab,kw. (99)
18     ("food and drug administration" adj3 data).ti,ab,kw. (157)
19     ("food and drug administration" adj3 website).ti,ab,kw. (46)
20     ("food and drug administration" adj3 web site).ti,ab,kw. (42)
21     trial$1 register.ti. (23)
22     trial$1 registers.ti. (13)
23     trial$1 registry.ti. (84)
24     trial$1 registries.ti. (36)
25     clinicaltrial$.ti. not (clinicaltrial$ or ISRCTN).si. (99)
26     current controlled trials.ti. (0)
27     (ictrp or mrct).ti. (2)
28     WHO portal.ti. (0)
29     publication bias.ti. (351)
30     european medicines agency.ti. (113)
31     (ema adj3 documents).ti,ab,kw. (4)
32     ((ema or emea) adj3 documents).ti,ab,kw. (10)
33     ((ema or emea) adj3 reports).ti,ab,kw. (17)
34     ((ema or emea) adj3 data).ti,ab,kw. (73)
35     ((ema or emea) adj3 website).ti,ab,kw. (21)
36     ((ema or emea) adj3 web site).ti,ab,kw. (4)
37     (european medicines agency adj3 documents).ti,ab,kw. (2)
38     (european medicines agency adj3 reports).ti,ab,kw. (6)
39     (european medicines agency adj3 data).ti,ab,kw. (12)
40     (european medicines agency adj3 website).ti,ab,kw. (18)
41     (european medicines agency adj3 web site).ti,ab,kw. (1)
42     licensing document*.ti,ab,kw. (5)
43     licensing application*.ti,ab,kw. (19)
44     (published adj3 (unpublished or "not published")).ab,kw. (3327)
45     (spontaneous report* and published).ti,ab,kw. (105)
46     ((differ* or compare or compared or compares or comparing comparison* or impact or value or use* or versus) adj3
(unpublished or "not published")).ab. (182)
47     *"Publication Bias"/ (885)
48     or/1-47 (6987)
49     (adverse adj2 (interaction$ or response$ or effect$ or event$ or reaction$ or outcome$)).ti,ab,kw. (270913)
50     side effect$.ti,ab,kw. (185122)
51     (unintended adj2 (interaction$ or response$ or effect$ or event$ or reaction$ or outcome$)).ti,ab,kw. (940)
52     (unintentional adj2 (interaction$ or response$ or effect$ or event$ or reaction$ or outcome$)).ti,ab,kw. (155)
53     (unwanted adj2 (interaction$ or response$ or effect$ or event$ or reaction$ or outcome$)).ti,ab,kw. (4430)
54     (unexpected adj2 (interaction$ or response$ or effect$ or event$ or reaction$ or outcome$)).ti,ab,kw. (4498)
55     (undesirable adj2 (interaction$ or response$ or effect$ or event$ or reaction$ or outcome$)).ti,ab,kw. (5938)
56     (serious adj2 (interaction$ or response$ or effect$ or event$ or reaction$ or outcome$)).ti,ab,kw. (26869)
57     (toxic adj2 (interaction$ or response$ or effect$ or event$ or reaction$ or outcome$)).ti,ab,kw. (42865)
58     (adrs or ades).ti,ab,kw. (3055)
59     drug safety.ti,ab,kw. (2906)
60     (drug surveillance or ((postmarketing or post marketing) adj2 surveillance)).ti,ab,kw. (2448)
61     product surveillance.ti,ab,kw. (29)
62     drug monitoring.ti,ab,kw. (6052)
63     tolerability.ti,ab,kw. (34536)
64     (harm or harms or harmful).ti,ab,kw. (66861)
65     treatment emergent.ti,ab,kw. (2307)
66     (iatrogenic or iatrogenesis).ti,ab,kw. (22654)
67     complication$.ti,ab,kw. (641336)
68     toxicity.ti,ab,kw. (267980)
69     pharmacovigilance.ti,ab,kw. (2559)
70     drug withdrawal*.ti,ab,kw. (2987)
71     ae.fs. (1400645)
72     to.fs. (338566)
73     co.fs. (1648100)
74     Product Surveillance, Postmarketing/ (5845)
75     Adverse Drug Reaction Reporting Systems/ (5858)
76     pharmacovigilance/ (709)
77     Drug Monitoring/ (15018)
78     exp Drug Hypersensitivity/ (38850)
79     Iatrogenic Disease/ (13264)
80     exp "Drug-Related Side Effects and Adverse Reactions"/ (90916)
81     Abnormalities, Drug-Induced/ (13900)
82     exp Postoperative Complications/ (432552)
83     exp Intraoperative Complications/ (41637)
84     Safety-Based Drug Withdrawals/ (270)
85     or/49-84 (4218996)
86     48 and 85 (2387)
87     (2009* or 2010* or 2011* or 2012* or 2013* or 2014* or 2015*).ed. (6034905)
88     86 and 87 (1152)

#### Data extraction

Information will be collected on the interventions and adverse effects studied, the sources of published and unpublished data, and the outcome measures (such as effect size or number of cases) used to compare the information on adverse effects from studies with differing publication status.

#### Assessment of methodological quality

The following criteria will be used to assess the quality of the included evaluations;

1. Adequacy of search to identify unpublished and published versions: For instance, were at least two databases searched for the published versions? Incomplete searches will either give the impression of fewer published or unpublished sources in existence.
2. Blinding: Was there any attempt to blind the data extractor to which version was published or unpublished?
3. Data extraction: Was the data extraction checked or independently conducted when comparing adverse events between the different sources?
4. Definition of publication status: Were explicit criteria used to categorise or define unpublished studies, and how did the investigators verify that a particular dataset was genuinely unpublished? For example, unpublished data may consist of information obtained from the manufacturers or regulatory agencies. Conversely, a broader definition of ‘grey literature’ may include information from websites, dissertations, policy documents, research reports, and conference abstracts.
5. External Validity and Representativeness: Did the researchers select a broad-ranging sample of studies (in terms of size, diversity of topics and spectrum of adverse effects) which were reasonably reflective of current literature?
6. Confounding factors in relation to publication status: The results in published sources may differ from those of unpublished sources due to factors other than publication status, such as methodological quality, study design, type of participant, and type of intervention. For instance, did the groups of studies being compared share similar features, other than the difference in publication status? Did the unpublished sources have similar aims, designs and sample sizes as the published ones? If not, were suitable adjustments made for potentially confounding factors?

#### Analysis

For methodological evaluations that measured incidence rates or number of cases of adverse effects only a descriptive comparison will be presented. The proportion of data only available in unpublished format will be expressed as a proportion of all the data available (in terms of numbers of studies, number of patients and numbers of adverse effects).

If the reviews present risk ratios or odds ratio, a comparison of the magnitude of treatment effect will be sought from unpublished studies versus that of published studies. A forest plot will be presented of the unpublished and published studies. The number of agreements and disagreements in terms of overlapping confidence intervals will be calculated.

#### References

1. Chou R, Aronsonb N, Atkinsc D, Ismailad AS, Santaguidad P, Smith DH, et al. AHRQ Series Paper 4: Assessing harms when comparing medical interventions: AHRQ and the Effective Health-Care Program *J Clin Epidemiol* 2010;63:502-12.
2. **Golder S**, Loke YK, Bland M. [Unpublished data can be of value in systematic reviews of adverse effects: methodological overview.](http://www.ncbi.nlm.nih.gov/pubmed/20457510) *Journal of Clinical Epidemiology* 2010;63(10):1071-1081.
3. **Golder S**, Loke YK. Is there evidence for biased reporting of published adverse effects data in pharmaceutical industry-funded studies? *British Journal of Clinical Pharmacology* 2008:66(6)767-773.

**FIRST APPROVED**

All authors first approved this protocol on the 12^th^ May 2015

**ADMENDMENTS – August 2015**

1. Item 6 of the assessment of methodological quality was only used for those studies which compared unmatched published and unpublished data. The following item was used for matched studies instead “Matching and confirming versions of the same study: For instance, were explicit criteria for intervention, sample size, study duration, dose groups etc. used? Were study identification numbers used, or were links/citations taken from unpublished versions?”
