# Supplementary material for: Reporting of Adverse Events in Published and Unpublished Studies of Health Care Interventions: A Systematic Review
Source: PLoS Med. 2016 Sep 20;13(9):e1002127. doi: 10.1371/journal.pmed.1002127 (PMC5029817; doi:10.1371/journal.pmed.1002127)
Supplement: S3 Text — (DOCX) [file pmed.1002127.s011.docx]

###### Databases

CINAHL Plus

Cochrane Database of Systematic Reviews (CDSR) – methodology reviews only

Cochrane Methodology Register (CMR)

Conference Proceedings Citation Index (CPCI)

Embase

Health Management Information Consortium (HMIC)

MEDLINE

MEDLINE In Process

OpenGrey

Proquest Dissertations & Theses: UK & Ireland

Proquest Library Science and Library and Information Science & Technology Abstracts (LISTA)

PsycINFO

Science Citation Index (SCI)

Scopus

Zetoc

###### Internet Search Engines

Google

Google Scholar

###### Handsearching of Journals

Drug Safety

Pharmacoepidemiology and Drug Safety

###### Hansearching of Bibliographies

Arber M, Cikalo M, Glanville J, Lefebvre C, Varley D, Wood H. Annotated bibliography of published studies addressing searching for unpublished studies and obtaining access to unpublished data. York: York Health Economics Consortium; 2013.

###### Handsearching of Conference Proceedings

Cochrane Colloquium
