# Supplementary material for: Reporting of Adverse Events in Published and Unpublished Studies of Health Care Interventions: A Systematic Review
Source: PLoS Med. 2016 Sep 20;13(9):e1002127. doi: 10.1371/journal.pmed.1002127 (PMC5029817; doi:10.1371/journal.pmed.1002127)
Supplement: S4 Text — (DOCX) [file pmed.1002127.s012.docx]

Database: Ovid MEDLINE(R) In-Process & Other Non-Indexed Citations and Ovid MEDLINE(R) <1946 to Present>

Original Search Strategy:

--------------------------------------------------------------------------------

1 unpublished.ti. (629)

2 "not published".ti. (8)

3 grey literature.ti. (27)

4 gray literature.ti. (6)

5 (clinical study report* or clinical trial report*).ti,ab,kw. (373)

6 regulatory website*.ti,ab,kw. (8)

7 regulatory web site*.ti,ab,kw. (2)

8 regulatory report*.ti,ab,kw. (39)

9 regulatory data.ti,ab,kw. (88)

10 regulatory document*.ti,ab,kw. (139)

11 (fda adj3 document*).ti,ab,kw. (125)

12 (fda adj3 reports).ti,ab,kw. (164)

13 (fda adj3 data).ti,ab,kw. (336)

14 (fda adj3 website).ti,ab,kw. (66)

15 (fda adj3 web site).ti,ab,kw. (45)

16 ("food and drug administration" adj3 document*).ti,ab,kw. (58)

17 ("food and drug administration" adj3 reports).ti,ab,kw. (99)

18 ("food and drug administration" adj3 data).ti,ab,kw. (157)

19 ("food and drug administration" adj3 website).ti,ab,kw. (46)

20 ("food and drug administration" adj3 web site).ti,ab,kw. (42)

21 trial$1 register.ti. (23)

22 trial$1 registers.ti. (13)

23 trial$1 registry.ti. (84)

24 trial$1 registries.ti. (35)

25 clinicaltrial$.ti. not (clinicaltrial$ or ISRCTN).si. (99)

26 current controlled trials.ti. (0)

27 (ictrp or mrct).ti. (2)

28 WHO portal.ti. (0)

29 publication bias.ti. (348)

30 european medicines agency.ti. (113)

31 ((ema or emea) adj3 document*).ti,ab,kw. (15)

32 ((ema or emea) adj3 reports).ti,ab,kw. (16)

33 ((ema or emea) adj3 data).ti,ab,kw. (73)

34 ((ema or emea) adj3 website).ti,ab,kw. (21)

35 ((ema or emea) adj3 web site).ti,ab,kw. (4)

36 (european medicines agency adj3 document*).ti,ab,kw. (5)

37 (european medicines agency adj3 reports).ti,ab,kw. (6)

38 (european medicines agency adj3 data).ti,ab,kw. (12)

39 (european medicines agency adj3 website).ti,ab,kw. (18)

40 (european medicines agency adj3 web site).ti,ab,kw. (1)

41 licensing document*.ti,ab,kw. (5)

42 licensing application*.ti,ab,kw. (19)

43 (published adj3 (unpublished or "not published")).ab,kw. (3328)

44 (spontaneous report* and published).ti,ab,kw. (105)

45 ((differ* or compare or compared or compares or comparing or comparison* or impact or value or use* or versus) adj3 (unpublished or "not published")).ab. (222)

46 *"Publication Bias"/ (885)

47 (or/1-30) or (or/31-46) (7223)

48 (adverse adj2 (interaction$ or response$ or effect$ or event$ or reaction$ or outcome$)).ti,ab,kw. (270922)

49 side effect$.ti,ab,kw. (185127)

50 (unintended adj2 (interaction$ or response$ or effect$ or event$ or reaction$ or outcome$)).ti,ab,kw. (940)

51 (unintentional adj2 (interaction$ or response$ or effect$ or event$ or reaction$ or outcome$)).ti,ab,kw. (156)

52 (unwanted adj2 (interaction$ or response$ or effect$ or event$ or reaction$ or outcome$)).ti,ab,kw. (4433)

53 (unexpected adj2 (interaction$ or response$ or effect$ or event$ or reaction$ or outcome$)).ti,ab,kw. (4492)

54 (undesirable adj2 (interaction$ or response$ or effect$ or event$ or reaction$ or outcome$)).ti,ab,kw. (5938)

55 (serious adj2 (interaction$ or response$ or effect$ or event$ or reaction$ or outcome$)).ti,ab,kw. (26885)

56 (toxic adj2 (interaction$ or response$ or effect$ or event$ or reaction$ or outcome$)).ti,ab,kw. (42872)

57 (adrs or ades).ti,ab,kw. (3054)

58 drug safety.ti,ab,kw. (2901)

59 (drug surveillance or ((postmarketing or post marketing) adj2 surveillance)).ti,ab,kw. (2447)

60 product surveillance.ti,ab,kw. (29)

61 drug monitoring.ti,ab,kw. (6050)

62 tolerability.ti,ab,kw. (34533)

63 (harm or harms or harmful).ti,ab,kw. (66889)

64 treatment emergent.ti,ab,kw. (2303)

65 (iatrogenic or iatrogenesis).ti,ab,kw. (22653)

66 complication$.ti,ab,kw. (641474)

67 toxicity.ti,ab,kw. (267972)

68 pharmacovigilance.ti,ab,kw. (2556)

69 drug withdrawal*.ti,ab,kw. (2987)

70 ae.fs. (1400999)

71 to.fs. (338639)

72 co.fs. (1648408)

73 Product Surveillance, Postmarketing/ (5846)

74 Adverse Drug Reaction Reporting Systems/ (5860)

75 pharmacovigilance/ (710)

76 Drug Monitoring/ (15022)

77 exp Drug Hypersensitivity/ (38855)

78 Iatrogenic Disease/ (13265)

79 exp "Drug-Related Side Effects and Adverse Reactions"/ (90937)

80 Abnormalities, Drug-Induced/ (13900)

81 exp Postoperative Complications/ (432670)

82 exp Intraoperative Complications/ (41657)

83 Safety-Based Drug Withdrawals/ (270)

84 or/48-83 (4219695)

85 47 and 84 (2475)

86 (2009* or 2010* or 2011* or 2012* or 2013* or 2014* or 2015*).ed. (6049653)

87 85 and 86 (1198)
